# Affinity purification with metabolomic and proteomic analysis unravels diverse roles of nucleoside diphosphate kinases

**DOI:** 10.1093/jxb/erx183

**Published:** 2017-06-06

**Authors:** Marcin Luzarowski, Monika Kosmacz, Ewelina Sokolowska, Weronika Jasińska, Lothar Willmitzer, Daniel Veyel, Aleksandra Skirycz

**Affiliations:** Max Planck Institute of Molecular Plant Physiology, Am Mühlenberg, Potsdam-Golm, Germany

**Keywords:** Affinity purification, *Arabidopsis thaliana*, glutathionylation, metabolites, NDPK, plant, protein–metabolite interactions, protein–protein interactions

## Abstract

Interactions between metabolites and proteins play an integral role in all cellular functions. Here we describe an affinity purification (AP) approach in combination with LC/MS-based metabolomics and proteomics that allows, to our knowledge for the first time, analysis of protein–metabolite and protein–protein interactions simultaneously in plant systems. More specifically, we examined protein and small-molecule partners of the three (of five) nucleoside diphosphate kinases present in the Arabidopsis genome (NDPK1–NDPK3). The bona fide role of NDPKs is the exchange of terminal phosphate groups between nucleoside diphosphates (NDPs) and triphosphates (NTPs). However, other functions have been reported, which probably depend on both the proteins and small molecules specifically interacting with the NDPK. Using our approach we identified 23, 17, and 8 novel protein partners of NDPK1, NDPK2, and NDPK3, respectively, with nucleotide-dependent proteins such as actin and adenosine kinase 2 being enriched. Particularly interesting, however, was the co-elution of glutathione *S*-transferases (GSTs) and reduced glutathione (GSH) with the affinity-purified NDPK1 complexes. Following up on this finding, we could demonstrate that NDPK1 undergoes glutathionylation, opening a new paradigm of NDPK regulation in plants. The described results extend our knowledge of NDPKs, the key enzymes regulating NDP/NTP homeostasis.

## Teaser

We demonstrate that affinity purification is suited for analysing protein–small molecule interactions. Analysis of protein–protein–small molecule complexes of Arabidopsis nucleoside diphosphate kinases gave new insight into their function and regulation.

## Introduction

Interactions between organic molecules (proteins, nucleic acids, and metabolites) regulate metabolic and signalling processes by acting as biological switches. Although general physicochemical principles governing these interactions are known, we are still far from having complete understanding of the interactome, particularly in the case of protein–metabolite interactions (PMIs). In addition to serving as substrates and cofactors for biochemical reactions, small molecules regulate vital biological processes by changing properties of their protein and/or nucleic acid partner(s) (Vassilev *et al.*, 2004; Granotier *et al.*, 2005), the classical example being the lactose operon in bacteria (Jacob and Monod, 1961). Identification of PMIs is therefore of fundamental importance for the understanding of cellular functions.

The main routes to identify protein interactors of a metabolite of choice (Jung and Kwon, 2015) include (i) affinity chromatography (Harding *et al.*, 1989); (ii) drug affinity-responsive target stability (DARTS) assay (Lomenick *et al.*, 2009); (iii) thermal proteome profiling (Pantoliano *et al.*, 2001); and (iv) chemo-proteomics (Manabe *et al.*, 2010).

Reverse strategies for finding small-molecule interactors, starting with the protein of interest, are often used in drug discovery and rely on recombinant proteins and chemically synthesized or natural compound libraries. While well applicable in *in vitro* situations, it is less suited for identifying *in vivo* signalling molecules. An alternative strategy exploits affinity purification (AP)- and MS-based metabolomics (Li and Snyder, 2011; Maeda *et al.*, 2013, 2014). This approach comprises the following steps: (i) preparation of transgenic lines expressing the protein of interest fused to an affinity tag, such as a G-protein; (ii) affinity purification of the tagged protein complexes; and (iii) analysis of the small molecules bound to the bait protein by LC/MS (Li and Snyder, 2011; Maeda *et al.*, 2014). Using this approach, Maeda *et al.* (2013) unravelled a plethora of novel LTP (lipid transfer protein)–lipid complexes. The main advantage of the AP–MS strategy is that the experiment is done in near-physiological conditions and thus is well suited for retrieving biologically relevant interactors. Herein, we extended the AP approach to plant cells and polar metabolites. To this end, we adapted a tandem affinity purification protocol customarily used to study protein–protein complexes in Arabidopsis cell cultures (Van Leene *et al.*, 2007). By doing so, we established a procedure that enables identification of the protein and small-molecule partners of the bait protein in one purification step.

As a case example, we chose to focus on nucleoside diphosphate kinases (NDPKs) in *Arabidopsis thaliana*. NDPKs are a group of highly conserved proteins that were originally classified as housekeeping enzymes, as they catalyse the transfer of a phosphoryl group between tri- and diphosphate nucleosides, with their bona fide ligands being CDP, UDP, GDP, and ATP (Parks and Agarwal, 1973). NDPK function began attracting more attention when *NDPK-A* was reported as the first metastasis suppressor gene in animals (Postel *et al.*, 1993). It is now clear that NDPKs have many roles outside nucleotide metabolism. Evidence that NDPK can phosphorylate proteins at histidine residues emerged from studies in mammalian cells (Attwood and Wieland, 2015). Moreover, NDPKs play a regulatory role via direct binding to the DNA and to a plethora of protein partners (Fukamatsu *et al.*, 2003; Thakur *et al.*, 2009). NDPKs can create direct or indirect complexes with cytoskeletal elements and have impact on their cellular functions (Menon and Schafer, 2013). For instance, NDPKs can promote activity of the GTPase dynamin and control polymerization of tubulin and the bacterial cytokinetic protein FtsZ (Jacobs and Huitorel, 1979; Mishra *et al.*, 2015) by influencing the exchange rate of GDP to GTP.

The activity of NDPKs is regulated by both protein and ligand partners. Thus, NDPKs are activated by a class of AMP-activated protein kinases (Onyenwoke *et al.*, 2012) and inhibited by 3',5'-cyclic AMP (3',5'-cAMP) (Anciaux *et al.*, 1997), which, at least in bacteria, occupies the usual nucleotide-binding site (Strelkov *et al.*, 1995). Eukaryotic NDPK-D was also reported to interact with lipids in the inner mitochondrial membrane. Such an interaction inhibits NDPK-D kinase activity but facilitates selective intermembrane lipid transfer (Schlattner *et al.*, 2013).

Plant NDPKs have been less studied as compared with their animal homologues. However, there is evidence that they display a similar functional versatility, which is again to a large extent dependent on their interaction partners. The Arabidopsis genome encodes five NDPKs, NDPK1–NDPK5 of which NDPK1, NDPK2, and NDPK3 are better characterized. Cytoplasmic NDPK1 was linked to oxidative stress and was found to interact with Catalase (CAT) 1, 2, and 3 using yeast two-hybrid assay (Fukamatsu *et al.*, 2003). NDPK1 co-purifies with G_1_/S cyclins in AP experiments (Van Leene *et al.*, 2010). NDPK2 localizes to the chloroplast and was described in relation to phytochrome and stress signalling (Choi *et al.*, 1999). Reported interactors include phytochromes PHYA and PHYB, numerous stress-related proteins such as mitogen-activated protein (MAP) kinases (MPK3 and MPK6) (Moon *et al.*, 2003), and CBL-interacting kinase SOS2 (Verslues *et al.*, 2007). In addition and similarly to bacteria, tobacco NDPKs were reported to bind to immobilized 3',5'-cAMP (Laukens *et al.*, 2001).

Given the (i) general importance of nucleotide pools for cellular homeostasis and the associated roles of NDPKs; (ii) multiple observations of NDPKs being part of protein–protein and protein–metabolite complexes, and (iii) different subcellular localization of the three NDPKs (NDPK1–NDPK3), a parameter that allows unlikely protein complex partners to be ruled out, we decided to study protein and metabolite interaction partners with an AP–MS protocol using the NDPK1, 2, and 3 proteins from *A. thaliana*.

We here apply and, to our knowledge, show for the first time the simultaneous analysis of polar metabolites and proteins from AP–MS experiments in plants, an approach that allows a comprehensive study of protein–protein–small molecule complexes.

## Materials and methods

### Cloning and generation of transgenic lines

**Fig. 1.**
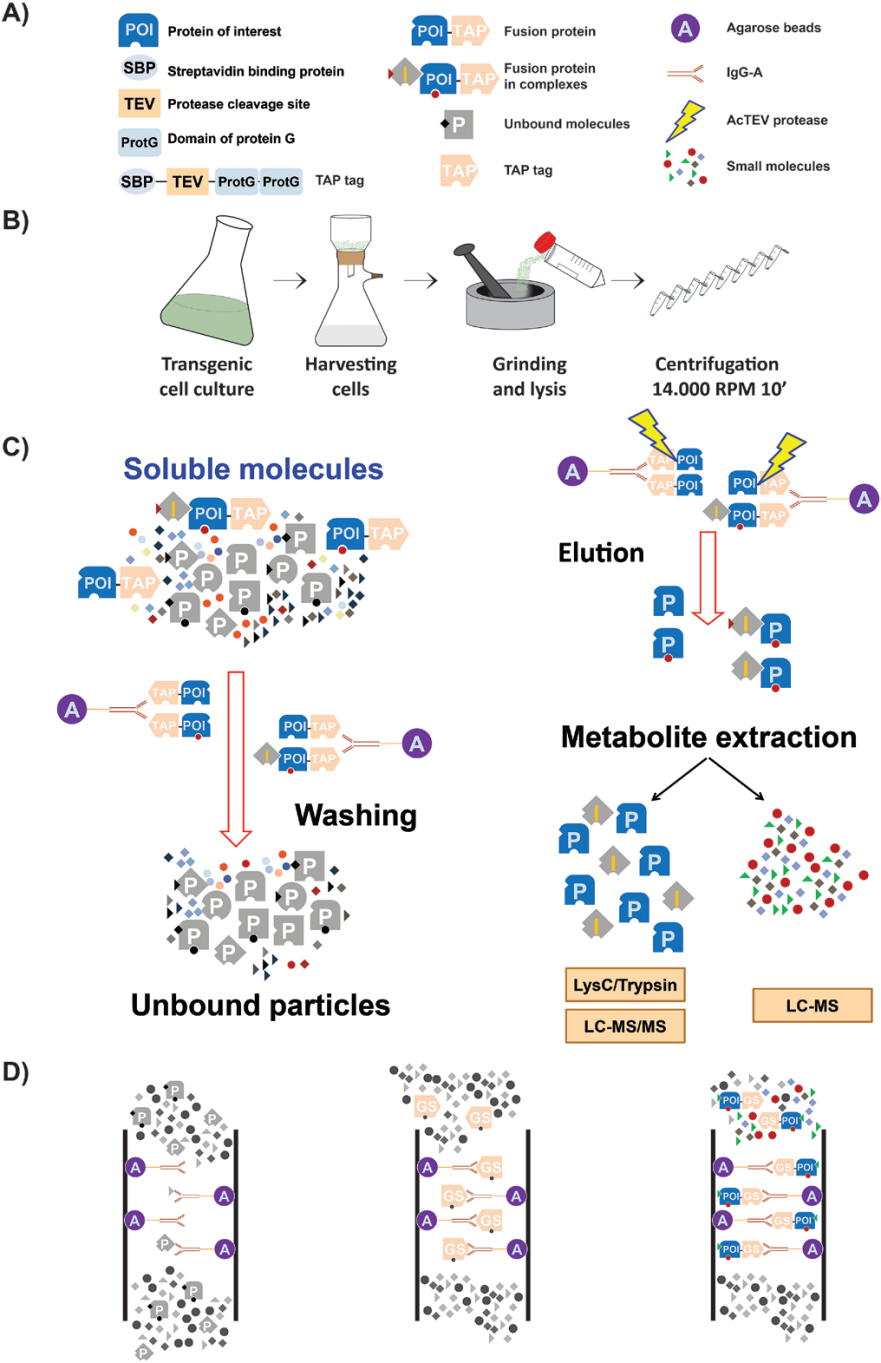
Overview of the AP–MS workflow used in our work. (A) TAP tag consists of two repeats of protein G, a streptavidin-binding domain, and a TEV protease cleavage site. The TAP tag can be fused to either the N- or C-terminus of the protein of interest. (B) Preparation of native cellular extract. (C) AP followed by simultaneous protein and metabolite extraction. (D) Unspecific binding of buffer compounds to agarose beads (blank control; left panel), unspecific binding of compounds of native cellular extract to the TAP tag (empty vector control; mid panel), and specific interactions between compounds of native cellular extract and fusion proteins (right panel).

The plasmids and primers used in this study are listed in Supplementary Tables S1 and S2 at *JXB* online. Cloning of the *35S:NDPK-TAP*, *35S:TAP-NDPK* and *EV-C*, *EV-N* constructs was performed using Gateway technology as described previously (Van Leene *et al.*, 2007). In brief, gene sequences coding proteins of interest were amplified using PCR and cloned into donor vectors (Supplementary Table S1, vector ID 1 and 2). Synthesized empty vector (EV) donor vectors (Invitrogen; Supplementary Table S1, vector ID 3 and 4) were linearized using FastDigest *Eco*RI and used as substrates for the BP recombination reaction (Supplementary Table S1, vector ID 2). Obtained donor vectors containing NDPK coding sequences or ATG/TAG codons were further used in the LR recombination reaction. Generated expression vectors encode fusion proteins: N- and C-terminally TAP-tagged NDPK1–NDPK3, ATG-TAP (EV-C), and TAP-TAG (EV-N) (Fig. 1A; Supplementary Table S1, vector ID 11–18). Western blot results using IgG antibody against the G-protein part of the TAP tag demonstrate proper expression of all the fusion proteins (Supplementry Fig. S1). *35S:NDPK-GFP* lines were obtained analogously using pENTR Directional TOPO and Gateway technologies (Supplementary Table S1, vector ID 19–21). Transformations of *Escherichia coli*, *Agrobacterium tumefaciens*, and cell cultures were performed as previously described (Van Leene *et al.*, 2007).

### Growth of Arabidopsis cell cultures

PSB-L Arabidopsis cells cultures were maintained as described by Van Leene *et al.* (2007). Cells were collected during logarithmic growth using a nylon mesh (wire diameter 34 µm, thickness 55 µm, open area 14%, Prosepa) and vacuum filtration (Fig. 1B). Dried cells were immediately frozen in liquid nitrogen.

### IgG affinity-based affinity purification

The AP protocol was adjusted from Van Leene *et al.* (2011) and Maeda *et al.* (2014). IgG Sepharose 6 Fast Flow beads (GE Healthcare) were equilibrated with lysis buffer (0.025 M Tris–HCl pH 7.5; 0.5 M NaCl; 0.015 M MgCl_2_; 0.5 mM DTT; 1 mM NaF; 1 mM Na_3_VO_4_; Protease Inhibitor Cocktail Sigma P9599). Plant cell material for the AP procedure was collected (as described above) from transgenic lines cultivated under identical growth conditions (Supplementary Table S3, line ID 1–8). Frozen material was pulverized to homogeneity in liquid nitrogen with a mortar and pestle. A 1 ml aliquot of lysis buffer was added per 1 g of pulverized material. Once thawed, samples were centrifuged at 4 °C, 20000 *g* for 10 min (Fig. 1B). On average, 3 g of plant material (corresponding to ~90 mg of total protein) and 100 µl of IgG beads were used per pull-down. Each purification (including empty vector control) was performed in triplicate, constituting three technical replicates. Binding was performed by 1 h incubation on a rotating wheel at 4 °C. Beads were transferred to a Mobicol ‘Classic’ filter (35 µM pore size, MoBiTec) and washed with 10 ml of wash buffer (0.025 M Tris–HCl pH 7.5; 0.5 M NaCl). The lower cap of the ‘Mobicol Classic’ was closed and 400 µl of elution buffer [10 mM Tris–HCl pH 7.5; 150 mM NaCl; 0.5 mM EDTA; E64 and 1 mM phenylmethylsulphonyl fluoride (PMSF)] containing 50 U of an improved version of the *Tobacco etch virus* (AcTEV) protease was added. Samples were incubated on a table shaker for 1 h at 16 °C with shaking at 1000 rpm. After 30 min, an additional 50 U of protease were added. Eluate was collected in a 2 ml Eppendorf tube. An additional 200 µl of elution buffer was passed over the beads and collected together with the previous eluate (Fig. 1C). In total, 600 µl of the eluate was used for metabolite and protein extraction. Importantly, the presence of the bait protein in the eluate was validated by both proteomics (Supplementary Table S4) and western blot analysis using antibodies (Abcam) against streptavidin-binding protein, which is a part of the tag left after TEV protease cleavage (Supplementary Fig. S1).

### Metabolite and protein extraction

Samples were extracted as described by Giavalisco *et al.* (2011). In brief, 600 µl of eluate was dried using a centrifugal evaporator and used as starting material. Proteins, lipids, and polar compounds were separated by a methyl-*tert*-butyl ether (MTBE)/methanol/water solvent system that separates molecules into a pellet, an organic, and an aqueous phase, respectively (Giavalisco *et al.*, 2011). For all replicates, an equal volume of aqueous phase was used for LC/MS analysis.

### Metabolomics

After MTBE extraction, the aqueous phase, containing semi-polar and polar compounds, was dried using a centrifugal evaporator and stored at –80 °C until LC/MS analysis. Small molecules were separated by ultraperformance liquid chromatography and analysed on an Exactive Orbitrap MS (Thermo Fisher Scientific) in positive and negative ionization modes as described in Giavalisco *et al.* (2011). Data processing, including peak detection and integration and removal of isotopic peaks and chemical noise, was performed using REFINER MS 7.5 (GeneData). An in-house database of chemical compounds was used to annotate obtained metabolic features (*m/z* at a given retention time) allowing 10 ppm and 0.15 min deviation from the reference compound mass and retention time, respectively. This approach led to annotation of eluted metabolites to a single match.

### Proteomics: sample preparation

Proteomic analysis was based on previous work of Olsen *et al.* (2004) and technical manual TM390 Trypsin/Lys-C Mix, Promega. In principle, protein fractions were subjected to enzymatic digestion prior to LC/MS/MS analysis. Protein pellets derived from metabolite extraction were dissolved in 40 mM ammonium bicarbonate (40 mM AmBic buffer) containing 6 M urea/2 M thiourea, pH 8 (Olsen *et al.*, 2004). Protein concentration was determined with the Bradford assay (Bradford, 1976). A 100 µg aliquot of protein in 46 µl of denaturation buffer (AmBic buffer pH 8.0, 2 M thiourea, 6 M urea) was treated with 2 µl of reduction buffer (50 mM DTT) and incubated for 30 min at room temperature. Then, 2 µl of alkylation buffer (150 mM iodoacetamide) was added to the sample and the mixture was incubated for 20 min at room temperature in the dark. Next, 30 µl of 40 mM AmBic buffer and 20 µl of LysC/Trypsin Mix were added and the sample was incubated for 4 h at 37 °C. After that, samples were diluted with 300 µl of 40 mM AmBic buffer and incubation continued at 37 °C overnight.

Samples were acidified with 2% trifluoroacetic acid (TFA) to pH <2 and proteins were desalted using Finisterre C18 SPE columns (Teknokroma™) as follows. The column was washed with 1 ml of 100% MeOH, 1 ml of 80% acetonitrile, 0.1% TFA (water solution), and equilibrated with 2 × 1 ml of 0.1% TFA (water solution). Samples were loaded on the columns; tubes were further washed with 200 µl of 0.1% TFA and loaded on the columns. Columns were washed with 2 × 1 ml of 0.1% TFA and peptides were slowly eluted with 800 µl of 60% acetonitrile, 0.1% TFA. Peptides were dried using a centrifugal evaporator and stored at –80 °C.

### Proteomics: LC/MS/MS analysis

To analyse peptide samples, we used an LC/MS system consisting of nano liquid chromatography (Proxeon EASY-nLC 1000, Thermo Fisher Scientific) with a reversed-phase column (C18, Acclaim PepMap RLSC, 75 µm, 15 cm, Thermo Fisher Scientific) connected to a Q Exactive Orbitrap Plus MS (Thermo Fisher Scientific).

Dried peptides were re-suspended in 50 µl of buffer A [3% v/v acetonitrile (ACN), 0.1% v/v formic acid]. Samples of 3 µl were separated by reverse-phase nano liquid chromatography using buffer A and buffer B (63% v/v ACN, 0.1% v/v formic acid). The gradient ramped from 3% ACN to 15% ACN over 20 min, then to 30% ACN in 10 min, followed by a 10 min washout with 60% ACN. The flow rate was 300 nl min^–1^ and the column was equilibrated with 5 µl of buffer A in between samples. The MS was run using a data-dependent MS/MS method with the following settings: full scans were acquired at a resolution of 70000, AGC target of 3 × 10^6^ ions, maximum injection time of 100 ms, and an *m/z* ranging from 300 to 1600. A maximum of 15 MS/MS scans were acquired per full scan (top 15) at a resolution of 17500, AGC target of 10^5^, maximum injection time of 100 ms, underfill ratio of 20%, with an isolation window of 1.6 *m/z* and an *m/z* ranging from 200 to 2000. Apex trigger was on (6–20 s) and a dynamic exclusion set to 15 s. Charge exclusion of charges 1 and >5 was on.

Data analysis was performed using MaxQuant software with the integrated Andromeda peptide search engine (Cox and Mann, 2008; Cox *et al.*, 2011) using default settings. The Uniprot database was downloaded on 15 March 2017 from http://www.uniprot.org/proteomes/UP000006548 as fasta (canonical and isoform) with all protein entries (33037), last modified 18 December 2016. The search also included the contaminant database (ftp://ftp.thegpm.org/fasta/cRAP). Peptides with at least seven amino acids were taken into account, with both the peptide and protein false discovery rate (FDR) set to 1% (see Supplementary Table S5 for ‘parameters.txt’ output file of MaxQuant analysis). Detailed information about all identified protein groups, including intensities, number of unique peptides, and score, is included in Supplementary Table S4 (see also Supplementary Table S6 and Supplementary Fig. S2 for a general overview of data and replicate quality).

Identified protein groups with less than two unique peptides and present at least in one technical replica of EV and/or blank controls were excluded from the list of potential interactors. Presence/absence of proteins was determined based on protein raw intensity (qualitative analysis). SUBAcon (consensus) location information was used to define subcellular targeting of protein partners (Tanz *et al.*, 2012; Hooper *et al.*, 2014).

Raw proteomics data were deposited in Pride.

### Confocal laser scanning microscopy (CLSM)

*35S:GFP*, *35S:NDPK1-GFP*, *35S:NDPK2-GFP*, and *35S:NDPK3-GFP* transgenic cell culture lines were analysed with a DM6000B/SP5 confocal laser scanning microscope (Leica Microsystems).

### *S*-Glutathionylation assay

*S*-Glutathionylation assay was performed using *35S:TAP-NDPK1* (Supplementary Table S3, line ID 2) transgenic plant cell cultures. A 24 g aliquot of plant cell material (3 g per one technical/conditional replicate) was collected, pulverized, and lysed as described above. To enrich for the NDPK1 protein, soluble extract was incubated with IgG–Sepharose beads as described above. Beads were washed to remove traces of DTT. An oxidation step using 500 µl of oxidizing agents was performed as follows. Samples, in two technical replicates, were treated with (i) 1 mM diamide and 1 mM GSH (reduced glutathione); (ii) 2.5 mM GSSG (oxidized glutathione); or (iii) 1 mM H_2_O_2_ and 1 mM GSH. Negative control (iv) was performed by the addition of 1 mM GSH. Samples were incubated for 1 h at room temperature in the dark. Oxidizing agents and GSH were separated from the beads by brief centrifugation. Subsequently, 400 µl of wash buffer (0.025 M Tris–HCl pH 7.5; 0.5 M NaCl, 2% SDS) was added to the beads, and proteins were released by denaturation in 95 °C for 10 min, followed by brief centrifugation. Eluate was collected in a 1.5 ml Eppendorf tube. An additional 200 µl of elution buffer was passed over the beads and collected together with the previous eluate. In total, 600 µl of eluate was used for protein precipitation by 80% acetone, for 20 h at –20 °C. On the following day, samples were centrifuged at 20000 *g* for 20 min, and pellets were washed with 80% acetone and centrifuged again at 20000 *g* for 20 min. The obtained pellets were re-suspended in denaturation buffer (AmBic pH 8.0, 2 M thiourea, 6 M urea), treated directly with alkylation buffer (150 mM iodoacetamide) while omitting the reduction step (to not reduce disulphide bonds between cysteine residues and glutathione), and prepared for MS measurements as described above. MS measurements were performed as described above. To analyse cysteine modifications of NDPK1 peptides, glutathionylation can be detected by a corresponding 305.11 Da increase in molecular mass. Raw data were analysed by REFINER MS 10.0 (GeneData). Protein identification was done with Mascot Daemon (Matrix Science) using the Ara_UniProt_2016 database, allowing up to two missed cleavages and 5 ppm peptide and 10 ppm MS/MS tolerance, respectively. Fixed modifications were excluded and variable modifications such as acetylation, glutathionylation, oxidation, and phosphorylation were allowed. MS/MS spectra as well as details of identification are presented in Supplementary Fig. S3A–H.

Glutathionylation efficiency was calculated as the ratio of the intensity of the glutathionylated form of the peptide and the sum of intensity of the carbamidomethylated and glutathionylated forms of the peptide (GLIGEVICR and GLIGEVICRFEK). Data represent average glutathionylation efficiency of two peptides, *n*=2.

### Protein thermal stability measurements

NDPK1 recombinant protein was obtained from a commercial supplier (MyBioSource) and stored in 20 mM Tris–HCl pH 8.0; 0.5 M NaCl; 20% glycerol. ATP, adenylosuccinate (ACA), and IMP stocks were prepared in water, while cAMP and cGMP were prepared in Tris buffer (20 mM Tris–HCl pH 8.0; 0.5 M NaCl). In accordance, NDPK1 was diluted to a final concentration of 2 µM using either water or binding buffer. Capillaries were loaded into the Prometheus NT.48 (Nanotemper). Unfolding was detected during heating in a linear thermal ramp (2 °C min^–1^, 30–90 °C) with an excitation power of 60–90%. Temperature-dependent protein unfolding was determined from changes in tryptophan and tyrosine fluorescence at emission wavelengths of 350 nm and 330 nm. Melting temperatures were determined by detecting the maximum of the first derivative of the fluorescence ratios (*F*350 nm/*F*330 nm) as described earlier (Martin *et al.*, 2014).

### NDPK enzymatic assay

The NDPK1 activity assay was adapted from Agarwal *et al.* (1978), whereby the formation of ADP from ATP in a coupled pyruvate kinase–lactate dehydrogenase system is measured spectrophotometrically. TDP was used as a NDPK1 substrate and phosphate group acceptor (Agarwal *et al.*, 1978). The rate of NADH oxidation was recorded by measuring the decrease in absorbance at 340 nm in an H1 Biotek plate reader (Biotek Company).

## Results

### Affinity purification strategy to identify protein–metabolite interactions

*Arabidopsis thaliana (At)NDPK* genes were cloned into plant binary vectors (Van Leene *et al.*, 2011) in which the constitutive 35S promoter is driving the expression of either C- or N-terminally tagged NDPKs. This tandem affinity tag is composed of a double repeat of the G-protein, followed by a TEV protease cleavage site and a streptavidin-binding domain (Fig. 1A). It enables two rounds of purification, first with an IgG antibody and afterwards with streptavidin. However, similar to Maeda *et al.* (2014), we abandoned the streptavidin purification step as biotin used for the elution would interfere with the metabolic profiling by overloading the chromatogram. For analogous reasons we simplified the lysis, wash, and elution buffers by removing detergents and glycerol. After washing and following elution with the TEV protease, the samples were subjected to liquid–liquid solvent extraction to separate semi-polar and lipophilic metabolites from proteins which are found in the pellet (Giavalisco *et al.*, 2011). In this manner, a single pull-down can be used for both protein and small-molecule analysis (Fig. 1B, C).

To correct for unspecific binding of proteins and metabolites to the tag and/or the resin, but not to the target proteins (NDPK1–NDPK3) themselves, we prepared a negative list of molecules from control experiments that were not considered further. Therefore, proteomic and metabolomic analyses were performed on affinity-purified complexes using EV control lines (Fig. 1D). In this way 1293 proteins and 30 metabolites were identified (see Supplementary Tables S7 and S8 for a list of these metabolites and proteins). As a further control, a blank sample was used (i.e. solely lysis buffer) to exclude small-molecule contaminants coming from chemicals and labware. The main molecules detected here were protease inhibitors, azelaic acid, and suberic acid, the latter two used, among others, to produce plastics and reagents. All proteins and small molecules found in either the blank or the empty vector control were added to the negative list.

To determine next both metabolites and proteins that most probably represent true interaction partners of the NDPK proteins, we applied the following criteria: (i) absent from the negative list (see above); and (ii) present in both the N- and C-tagged NDPK pull-down samples.

### NDPK1–NDPK3 localize in different subcellular compartments

As described above, we filtered the AP–MS data against unspecific bindings and blank controls. However, another filter that could be applied to this type of data is based on the supposition that true *in vivo* complexes are composed of proteins present in the same subcellular compartment. As reported data on the subcellular localization of the three NDPKs are to some extent controversial, we decided to retest their organellar distribution. To this end, transgenic Arabidopsis PSB-L cells expressing one of the *NDPK* genes as a C-terminal fusion with green fluorescent protein (GFP) under the control of a 35S *Cauliflower mosaic virus* (CaMV) promoter were analysed using CLSM.

**Fig. 2.**
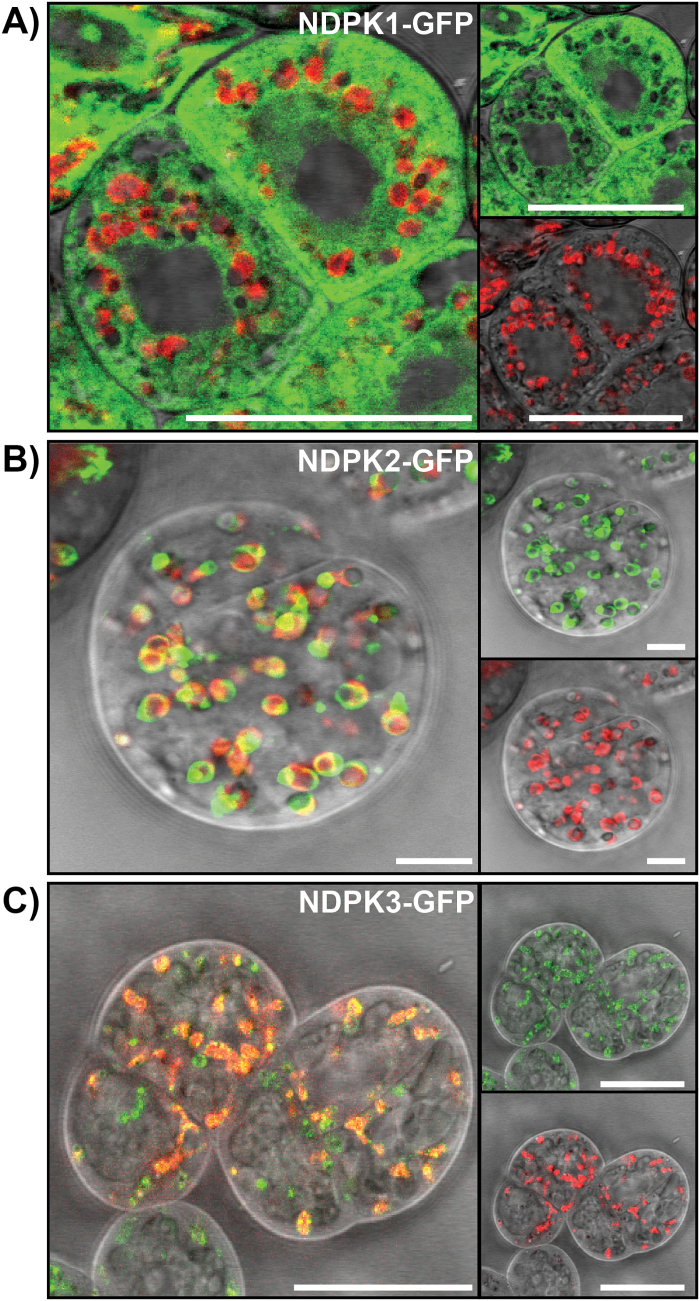
Subcellular localization of NDPK–GFP fusion proteins. CLSM images of 6- to 8-day-old NDPK–GFP Arabidopsis PSB-L transgenic cell cultures. (A) NDPK1–GFP signal is observed in the cytoplasm. (B) NDPK2–GFP signal overlaps with plastid autofluorescence, indicating chloroplastic localization. (C) NDPK3–GFP signal overlaps with plastid autofluorescence. Green (upper, right panel), signal of NDPK–GFP fusion protein; red (lower, right panel), plastid autofluorescence; grey, bright field. Scale bar=25 µm.

NDPK1 was previously reported to localize mainly in the cytosol (Dorion *et al.*, 2006). However, and despite the absence of an appropriate signal sequence, it was also described to be present in the nucleus and peroxisomes (Reumann *et al.*, 2009). A similar situation was described for the mammalian homologues NDPK-A and NDPK-B (Bosnar *et al.*, 2009). Nevertheless, and in agreement with Dorion *et al.*, our data showed predominant localization of AtNDPK1 in the cytosol (Fig. 2A).

Similar to AtNDPK1, the localization of AtNDPK2 is also disputed. Despite the presence of a putative plastid signal sequence, AtNDPK2 was reported to be localized to the nucleus and cytoplasm (Choi *et al.*, 1999). Later its localization was revisited, and it was demonstrated that AtNDPK2 is exclusively targeted to chloroplasts (Bölter *et al.*, 2007). In agreement with Bölter *et al.*, our data confirm that AtNDPK2 is exclusively localized in the chloroplast (Fig. 2B).

As to the subcellular localization of AtNDPK3, bioinformatic analysis suggested dual targeting to mitochondria and plastids (Dorion and Rivoal, 2015), which could indeed be confirmed experimentally. NDPK3 was demonstrated to localize in the intermembrane space of mitochondria (Sweetlove *et al.*, 2001) and in the thylakoid lumen of chloroplasts (Spetea *et al.*, 2004). In our experiments, using PSB-L cells, AtNDPK3 localized to chloroplasts and possibly also to mitochondria (Fig. 2C).

### Novel protein and metabolite partners of NDPKs

**Fig. 3.**
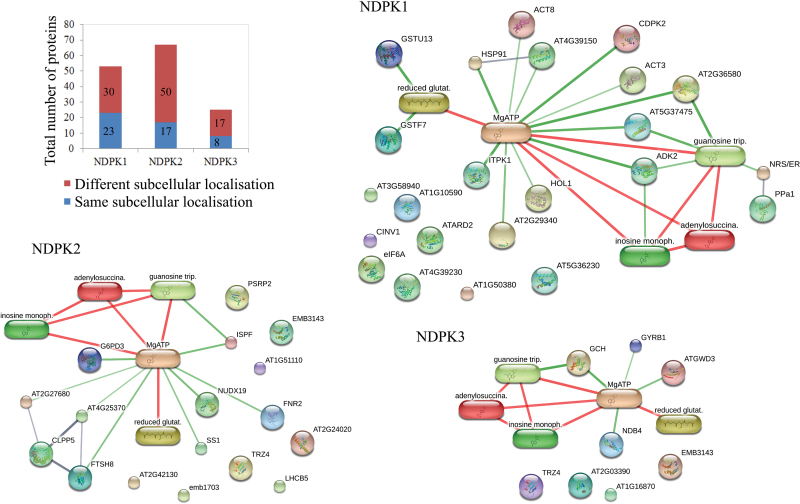
Proteins and small-molecule ligands pulled-down with NDPKs. The total number of protein partners co-purified with NDPK1–NDPK3 based on absence from the negative list but presence in the C- and N-tagged samples (see also Supplementary Tables S9 and S10). Blue indicates proteins that passed the subcellular localization filter: 23, 17, and 8 for NDPK1, 2, and 3, respectively. These, together with metabolites co-purified with NDPKs (glutathione, IMP, and adenylosuccinate) and nucleotide triphosphates directly related to NDPK function (ATP and GTP) were used to query the Stich database for protein–protein and protein–metabolite complexes. We used a minimum required interaction score of 0.5 and restricted our search to (i) experimentally, (ii) database-, and (ii) literature-reported associations. Subcellular protein localization was retrieved from the SUBA database (Tanz *et al.*, 2012).

Using the binding criteria of (i) absence from the negative list and (ii) presence in both the N- and C-tagged NDPK pull-down samples, we could identify 53, 67, and 25 putative protein partners for NDPK1, NDPK2, and NDPK3, respectively (Fig. 3; Supplementary Table S9). Furthermore, when applying an additional subcellular localization filter, and thus accepting only cytoplasmic proteins in the case of NDPK1, only plastidic proteins in the case of NDPK2, and plastidic and mitochondrial proteins in the case of NDPK3, the number of putative interactors was reduced to 23, 17, and 8, respectively (Fig. 3; Supplementary Table S9). The identified proteins are associated with diverse cellular processes, including response to oxidative stress [glutathione *S*-transferases (GSTs)], cytoskeleton formation (actin), protein folding and hydrolysis (heat-shock protein, Clp protease subunits), signal transduction (calcium-dependent protein kinase 11, inositol-tetrakisphosphate 1-kinase), and metabolism (starch synthase, glucose-6-phosphate 1-dehydrogenase 3).

With respect to small molecules, three metabolites (reduced glutathione, IMP, and ACA) were found specifically in the NDPK1 pull-down samples, but not in the EV control samples (Fig. 3; Supplementary Table S10). Glutathione and IMP were also co-purified with NDPK2, but not with NDPK3.

The 46 proteins and three metabolite interactors were queried against the Stitch database (Szklarczyk *et al.*, 2015) to look for reported metabolite–protein and protein–protein interactions (Fig. 3). Additionally, and with respect to the NDPK function, we included ATP and GTP in the query list. The main associations were as follows. IMP, ACA, and adenosine kinase 2 (ADK2) are present in the purine nucleotide cycle pathway. Glutathione is a principle ligand for GST. Twenty-three of the 50 proteins were associated with either ATP or GTP.

Furthermore, literature inspection of the protein complex data set showed that one protein which formed a complex with NDPKs based on our experiments, namely alkaline/neutral invertase CINV1, has also been described by Aryal *et al.* (2014) as a possible NDPK1 interaction partner (Fig. 3).

### Cys43 of NDPK1 is subjected to *S*-glutathionylation

As animal NDPKs were shown to be subjected to regulatory *S*-glutathionylation (Lee *et al.*, 2009), and as NDPK1 was co-purified with GST and glutathione (Fig. 3B), we decided to analyse whether or not Arabidopsis NDPK1 can be *S*-glutathionylated. We tested various oxidizing conditions. Recombinant NDPK1 protein was incubated with oxidized glutathione (GSSG) and the reduced form of glutathione (GSH) in the presence of either diamide or H_2_O_2_. NDPK1 was also treated with reduced GSH without addition of exogenous oxidizing agents. Glutathionylation was finally analysed by LC/MS/MS.

**Fig. 4.**
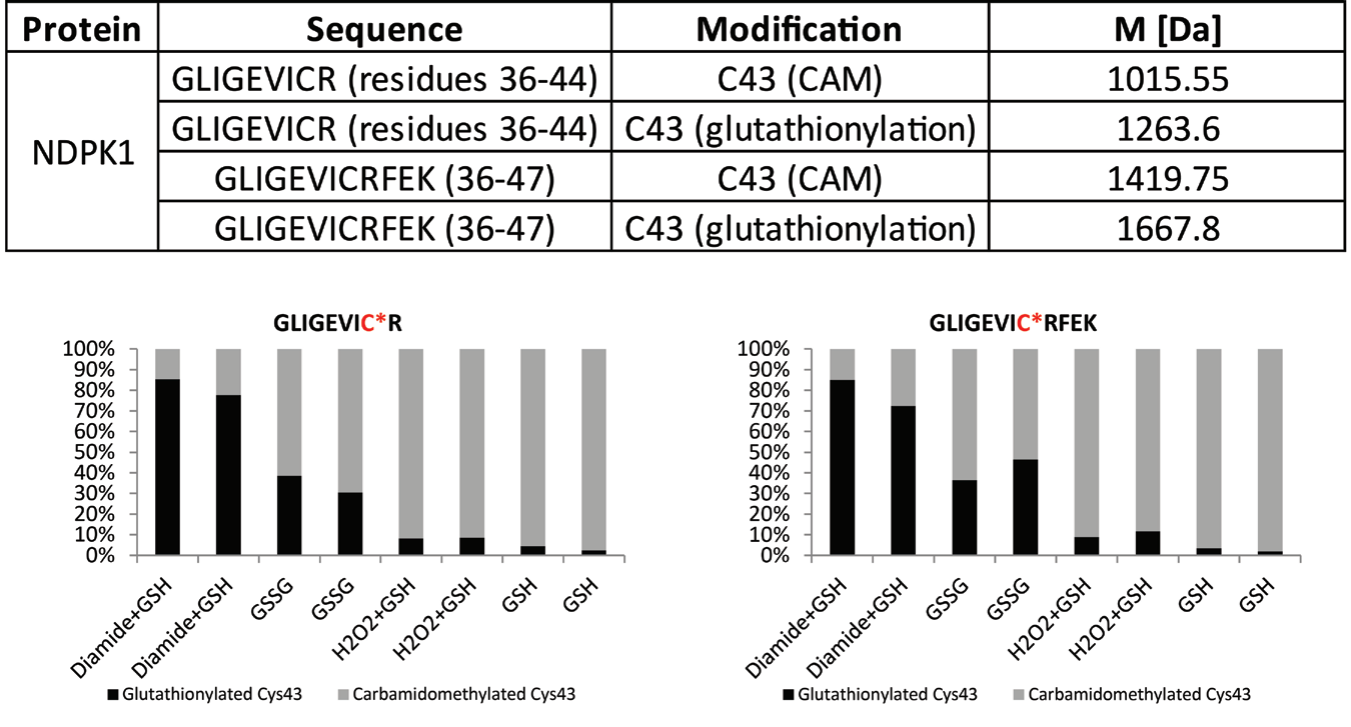
MS/MS detection of cysteine modifications of AtNDPK1. NDPK1 AP eluate sample was treated with different oxidizing agents: (i) 1 mM diamide and 1 mM GSH; (ii) 2.5 mM GSSG; (ii) 1 mM H_2_O_2_ and 1 mM GSH for 1 h at room temperature. Negative control was prepared by addition of 1 mM GSH. Afterwards free cysteine residues were blocked with carbamidomethyl (CAM) before tryptic digestion and MS analysis. The table shows identified peptide sequences containing modified cysteine. The lower panel demonstrates the efficiency of the singular treatments. The total amount of glutathionylated and carbamidomethylated Cys43 was set as 100%. All treatments were performed in duplicate. For further details see the Materials and methods.

Different oxidizing treatments led to clear glutathionylation of NDPK1 Cys43, whereas NDPK1 treated with GSH already showed low levels of glutathionylation (Fig. 4). In line with published data (Kosower and Kosower, 1995), diamide appears to be an efficient agent for promoting disulphide bond formation. Treatment with GSH and diamide led to glutathionylation of 80% of NDPK1 Cys43. GSSG and H_2_O_2_ were less efficient and led to glutathionylation of 38% of cysteine residues, whereas addition of the reduced form of glutathione led to modification of 9% of NDPK1 Cys43.

### NDPK1 interacts directly with cyclic nucleotides, but not with IMP and ACA

**Fig. 5.**
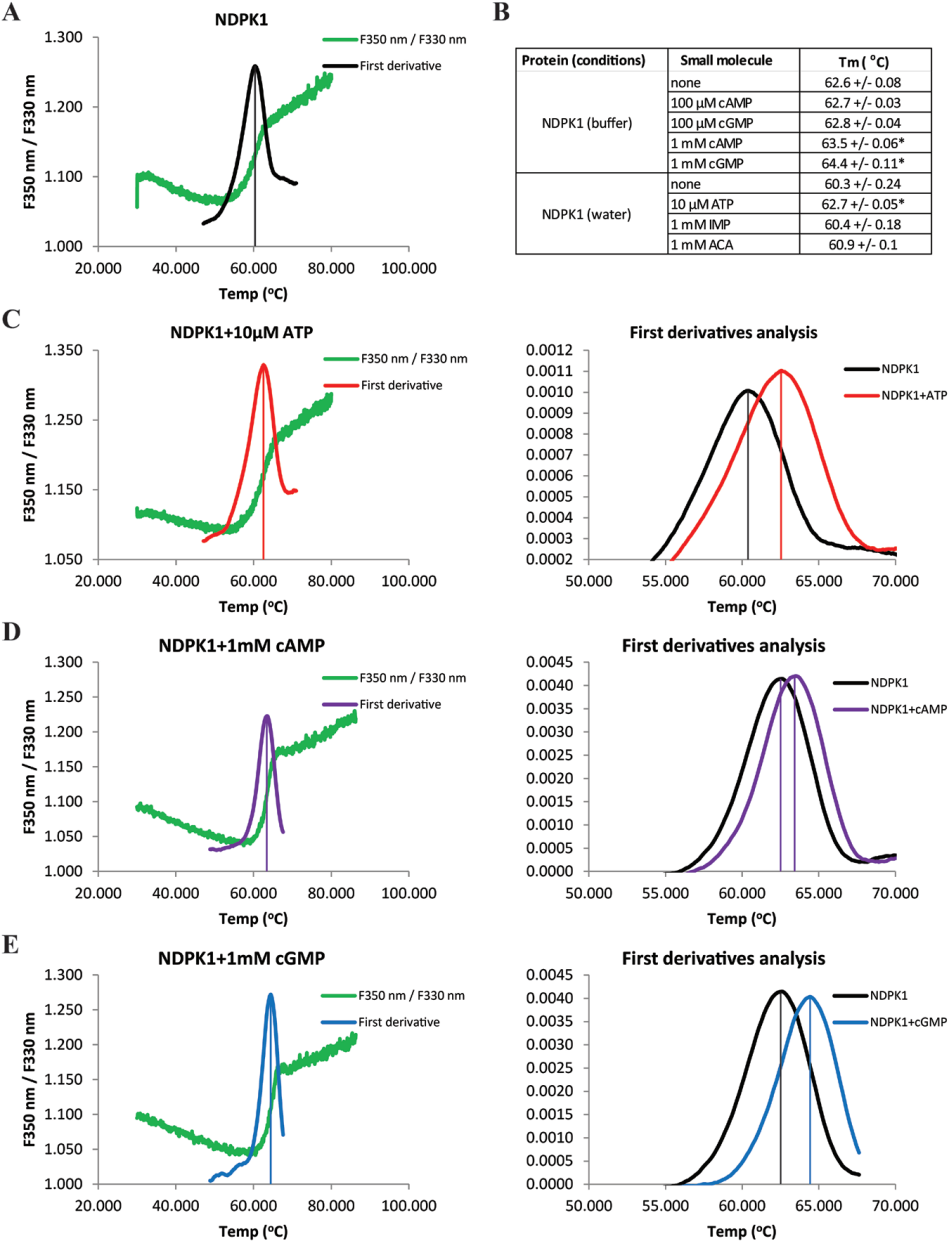
Thermal stability of recombinant NDPK1 protein in the presence of small-molecule ligands. Transition from the folded to unfolded state (melting) was calculated from changes in the ratio of *F*350 nm/*F*330 nm fluorescence measured across a temperature gradient (30–90 °C). (A, C–E) The fluorescence ratio (*F*350 nm/*F*330 nm) and the first derivative of the fluorescence ratio [Δ(*F*350 nm/*F*330 nm)/Δ*T*] as a function of temperature. Vertical lines indicate the melting temperature (*T*_m_) in the presence of different ligands. (C–E) Comparison of first derivatives obtained for NDPK1 alone and in the presence of ATP, cAMP, and cGMP. (B) Melting temperature measured for NDPK1 alone and in the presence of ligands. A change in the melting temperature is indicative of a binding event. Data represent the average±SD, *n*=3. * indicates *P*<0.01 in a Student *t*-test.

To investigate NDPK1 binding to IMP and ACA, retrieved in the AP experiments, we exploited a well-known phenomenon in which ligand binding affects the melting temperature (*T*_m_) of its protein partner (DeSantis *et al.*, 2012). Changes in the intrinsic fluorescence of tryptophan and tyrosine upon protein unfolding (melting) were followed to determine the *T*_m_ of recombinant NDPK1 in the absence (Fig. 5A) and presence of different concentrations of potential small-molecule ligands (Fig. 5B–E). As anticipated, ATP, a known NDPK1 substrate, stabilizes the protein already at a concentration of 10 μM (Fig. 5C). In contrast, neither IMP nor ACA had an effect on NDPK1 thermal stability, making them unlikely to be direct ligands of NDPKs (Fig. 5B). Prompted by published results (e.g. Laukens *et al.*, 2001), we also tested NDPK1 against cyclic nucleotides. 3',5'-cAMP and 3',5'-cGMP at 1 mM stabilized NDPK1, shifting the *T*_m_ by 0.9–1.8 °C (Fig. 5D, E).

**Fig. 6.**
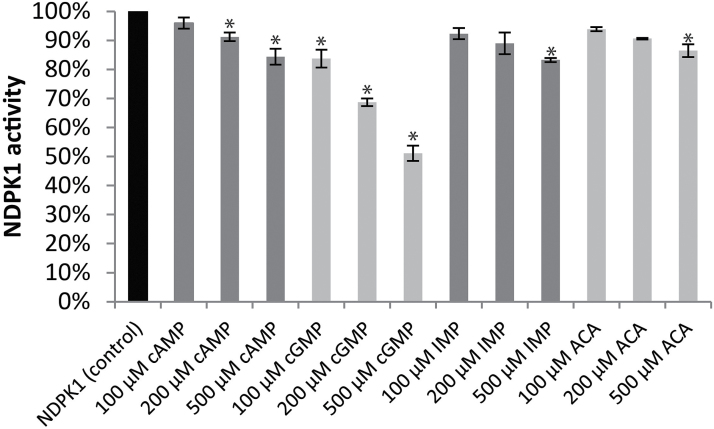
Activity of recombinant NDPK1 protein in the presence of small-molecule ligands measured using the pyruvate kinase–lactate dehydrogenase coupled assay. Measurements were performed with a fixed (0.004 M) TDP concentration and increasing amounts of small-molecule ligands. Percentage inhibition was calculated in relation to NDPK activity in the absence of the ligands. Data represent the average±SD, *n*=3. * indicates *P*<0.01 in a Student *t*-test.

To obtain independent evidence for the interaction between small molecules and NDPKs as suggested by the thermal stability assay, we performed enzymatic assays. The activity of the recombinant NDPK1 was measured in the presence of cAMP, cGMP, IMP, and ACA. The specific activity of NDPK1 in standard conditions was 12.1 U mg^–1^. Both cAMP and cGMP decreased the reaction speed of NDPK1 proportionally to the ligand concentration, cGMP being a stronger inhibitor (Fig. 6). Addition of 100, 200, and 500 µM cAMP lowered the specific activity of NDPK1 to 96, 91, and 84%, respectively, of the control, whereas addition of cGMP reduced the reaction speed to 84, 69, and 51% of the control. We did not observe a significant inhibitory effect of IMP or ACA, with the exception of a modest effect at the highest concentration (500 µM).

## Discussion

### AP–MS is—with some precautions—a suitable method for studying PMIs *in planta*

Herein we report, to our knowledge for the first time, the suitability of AP for PMI studies in plants. Our protocol enables the identification of putative protein and small-molecule partners of target proteins in a single experiment. The main advantage of this approach is that it captures interactions that occur in native plant lysates in near-physiological conditions in terms of interaction partner composition and concentration. When using Arabidopsis cell cultures it takes only 6–8 weeks to obtain stable transformants, with the affinity pull-down requiring merely 1 d.

The prominent drawback of affinity approaches is, however, the high rate of false positives. To correct for the background coming from unspecific binding of proteins and metabolites to, for example, agarose beads, we recommend using EV control lines and, in order to exclude contaminants coming from chemicals and labware, to introduce blank samples. By doing so, we excluded 1298 proteins and 30 metabolites found in the empty vector and/or blank samples as false positives. Experimenting with an alternative tag, introducing more stringent washing steps, and shortening incubation with the agarose beads can all be considered testable solutions to reduce the number of false positives. Alternatively, Maeda *et al.* (2014) performed an additional step of purification using size-exclusion chromatography. This additional step, however, makes the protocol considerably longer and labour-intensive, and dramatically decreases the number of samples that can be processed in parallel.

Another pertinent point relates to the fact that, unless working with isolated organelles, whole-cell lysates will contain mixtures of proteins and small molecules from different subcellular compartments (Aryal *et al.*, 2014), which may lead to the formation of artificial complexes absent *in vivo*. As our three Arabidopsis NDPKs share a high degree of homology (Cho *et al.*, 2004), their presence in a mixture of proteins and small molecules from different organelles might lead them to interact with the same set of proteins and metabolites (e.g. ADK2 or alkaline/neutral invertase CINV1), but the ‘true interactors’ will be secured by the same subcellular localization. NDPK1 is localized in the cytosol, NDPK2 in the chloroplast, and NDPK3 in the chloroplast and mitochondria. Thus proteins co-purifying with NDPK1 and NDPK2/NDPK3 should not have a major overlap. To correct for this artefact, we took the subcellular localization data into account. To illustrate: in the past, NDPK2 had been reported to interact with cytosolic proteins: PHYA, PHYB, and MAP kinases (Choi *et al.*, 1999; Moon *et al.*, 2003). However, these complexes were critically re-evaluated when NDPK2 was shown to localize to chloroplasts rather than the cytosol (Bölter *et al.*, 2007). Another thing to consider when working with cell lysates relates to the subcellular localization of the tagged proteins. To find interaction partners of NDPKs, we generated lines tagged in the N- and C-terminus, respectively. While C-terminal fusions should not have any detrimental effect, N-terminal fusions could disturb the organellar localization of NDPK2 and NDPK3 by masking the transit peptide. We chose to use N-terminal fusions nevertheless, reasoning that in the native lysate the true interaction, even if absent in the cell due to mislocalization, will form *de novo*.

Moreover, any affinity pull-down by its nature retrieves whole complexes, rendering the distinction between direct and indirect targets of the baited protein impossible. For instance, NDPK1 pulled-down retrieved glutathione but also GST, a bona fide receptor of glutathione. This is an advantage on the one hand, as it provides more information about the interactome as such. On the other hand, it requires follow-up experiments to obtain precise understanding of the complex topology. We reason that IMP and ACA, pulled-down together with the NDPKs, constitute indirect small molecule partners of the NDPKs by binding to NDPK protein partners, such as, for example, ADK2.

Note that the approach is also very much dependent on the sensitivity and the specificity of the proteomic and metabolomic pipelines used for analysis. For instance, in this particular study, we limited our small-molecule analysis to semi-polar compounds obtained by MTBE extraction. Though this enables us routinely to measure and annotate several hundred small molecules, many, including ATP, remain inaccessible.

Interactions between small molecules and proteins are considered relatively weak. Still, our study, alongside published work (Harding *et al.*, 1989), demonstrates that at least a subset of small molecule–protein complexes can be retrieved and studied, on a par with protein–protein complexes (Veyel *et al.*, 2014).

### Glutathione and cyclic nucleotides: potential small-molecule interaction partners provide new insight into NDPK1 regulation

Using our AP protocol, we could demonstrate that NDPK1 complexes with GST and glutathione, giving insight into NDPK regulation in plants. The established role of GSTs is the conjugation of the reduced form of glutathione to different substrates for the purpose of detoxification; yet some members of the GST family are involved in the *S*-glutathionylation of interacting protein partners (Townsend *et al.*, 2009). *S*-Glutathionylation modulates the function of the target protein and is involved in redox signalling mechanisms (Dalle-Donne *et al.*, 2009). We propose a working hypothesis in which AtNDPK1 would undergo *S*-glutathionylation under oxidative stress conditions. In the short term, *S*-glutathionylation would most probably inhibit NDPK activity; but ultimately *S*-glutathionylation would prevent irreversible cysteine oxidation during oxidative stress and thus preserve kinase activity until normal cellular ATP levels and the reducing environment are restored. In support of this, we could demonstrate that Cys43 of NDPK1 undergoes *S*-glutathionylation under oxidative conditions, making NDPK1 a likely target of redox signalling in plants. Our findings are supported by previously published work: Lee *et al.* (2009) reported that Nm23-H1, an animal homologue of AtNDPK1, is also regulated by redox signalling. In response to oxidative stress, Cys109 of Nm23-H1 is oxidized (including glutathionylation), inhibiting Nm23-H1 activity and metastatic suppressor properties (Lee *et al.*, 2009). Interestingly, NDPK1 can also undergo *S*-nitrosylation, a different redox modification of Cys43 (Fares *et al.*, 2011). Finally, in addition to being a subject of oxidative stress signalling, NDPK1 has been implicated in the oxidative stress response by complexing with CATs 1–3 (Fukamatsu *et al.*, 2003).

The connection between NDPKs and cyclic nucleotides was first reported in bacteria, when Strelkov *et al.* (1995) published the X-ray structure of the *Myxococcus xanthus* NDPK complexed with 3',5'-cAMP. They could show that cAMP occupies the same binding pocket as NDPK substrates, and by doing so inhibits enzyme activity, with a *K*_i_ of ~500 μM. Since then, NDPKs were repeatedly found in affinity pull-downs starting with cAMP and cGMP agarose resins in both animal and plant systems (Laukens *et al.*, 2001; Dubovskaya *et al.*, 2011). Using recombinant NDPK1, we could demonstrate that, similar to bacterial NDPK, the Arabidopsis enzyme binds cAMP and cGMP, leading to a reduction in enzyme activity. Similar to the bacterial counterpart, and at least *in vitro*, both binding and inhibition occurred in the mid to high micromolar range. *In vivo* concentrations of cyclic nucleotides are in the much lower, nanomolar range (e.g. Gehring, 2010). Still it is not uncommon that binding affinity measured *in vitro* would be very much different from the *in vivo* situation. More difficult to address is the fact that inside the cells cNMPs compete for the binding site with the much more abundant NDPs, the main argument against a physiological significance of NDKP–cNMP interactions. Therefore, the binding would only occur if we assume the existence of a local gradient of NDP and cNMP concentration that favours the latter. This is a hypothesis that we are not able to test using existing methods. It is, however, a tempting speculation: in animal cells, for instance, NDPKs were shown to stimulate cAMP production by a direct activation of G-proteins, resulting in such local cNMP maxima (Hippe *et al.*, 2007).

### Novel protein partners for the different NDPKs

When accepting only cytoplasmic proteins in the case of NDPK1, plastidic proteins in the case of NDPK2, and plastidic and mitochondrial proteins in the case of NDPK3, the number of putative proteins present in a complex with the different NDPKs was reduced to 23, 17, and 8 for NDPK1, NDPK2, and NDPK3, respectively.

The interactors found share no obvious functional similarity. However, as expected for enzymes that regulate the NTP/NDP balance, they are enriched in ATP/ADP- and GTP/GDP-dependent proteins. The most interesting interactors are discussed below. Note that independent validation of the protein–protein interactions (e.g. using a two-hybrid system) is recommendable if taking the project further.

Previous studies demonstrated that eukaryotic and bacterial NDPKs control polymerization of tubulin and bacterial cytokinetic protein, respectively, by mediating phosphorylation of the protein-bound GDP to GTP (Mishra *et al.*, 2015). Moreover, there is evidence that eukaryotic NDPKs create complexes with regulators of the actin cytoskeleton (reviewed by Snider *et al.*, 2015). Our results would endorse the presence of a similar mechanism in plants, as NDPK1 was found in a complex with actin, suggesting its involvement in cytoskeleton regulation.

Pyruvate kinase, together with ADK2, are also nucleotide-dependent proteins complexed with NDPK1. Pyruvate kinase is involved in the synthesis of pyruvate and ATP from phosphoenolpyruvate and ADP (Krebs and Eggleston, 1965), thus potentially serving as a direct donor of ATP for cytosolic NDPK1. ADK2 catalyses ATP-dependent phosphorylation of adenosine to monophosphates and, by regulating adenosine levels in the cell, affecting transmethylation (Moffatt *et al.*, 2000, 2002). We speculate that some of the reported interactions, namely NDPK1–pyruvate kinase, NDPK1–calcium-dependent protein kinase 11, or NDPK1–inositol-tetrakisphosphate 1-kinase 1, may serve to maintain local ATP levels essential for the enzymatic activity of co-eluted enzymes.

NDPK2 is the best characterized of the five Arabidopsis NDPKs. Its function is linked to light signalling, chlorophyll biosynthesis, and abiotic stress response (e.g. Ye *et al.*, 2016). Previous linking of NDPK2 to light signalling (reviewed by Dorion and Rivoal, 2015) is reinforced by our results that show interaction between NDPK2 and a component of the light-sensing machinery, specifically Chl *a*-*b*-binding protein CP26.

Our results putatively reveal a novel regulatory function for NDPK2 and NDPK3, namely protein hydrolysis, a function shared by 4 of the 23 proteins found together with NDPK2 and NDPK3 in our AP experiments: ATP-dependent Clp protease proteolytic subunit 5, ATP-dependent Clp protease ATP-binding subunit CLPT1, isoform 2 of Clp protease adaptor-protein ClpF (ClpF), and ATP-dependent zinc metalloprotease FTSH 8 (Kim *et al.*, 2009, 2015; Zhang *et al.*, 2010; Nishimura *et al.*, 2015). Regulation of the chloroplastic NTP pool by the plastid-specific NDPK2 and the dually targeted NDPK3 can serve as an important regulatory mechanism for protein hydrolysis, suggesting linkage between the levels of small molecules and, in this case, organelle-specific proteomes. Yet, it is worth considering that NDPKs might also be substrates for the Clp protease system, since NDPK3’s interaction partner, ClpF, was proposed as a part of a recognition complex that marks substrates targeted for hydrolysis.

Lastly remains the issue of our failure, with the exception of alkaline/neutral invertase, to find already reported NDPK1–NDPK3 protein partners, most notably CATs, MPKs, PHYA and PHYB, SOS2, and cyclins (Choi *et al.*, 1999; Fukamatsu *et al.*, 2003; Moon *et al.*, 2003; Van Leene *et al.*, 2010). This can be explained by a number of reasons. CAT1, CAT2, and MPK4, for instance, were on the ‘negative list’ as they co-purified with the tag alone. Cyclins found together with NDPK1 in the TAP experiments were most probably missing in our identification due to a different starting material. While Van Leene *et al.* (2010) used activly dividing cells, we collected stationary phase cells, with low or no expression of cell cycle proteins.

In summary, the multitude of NDPK protein partners reported here, but also by others, supports the involvement of NDPKs in a myriad of biological processes connected to nucleotide homeostasis. Additional functions such as, for example, histidine protein kinase activity clearly needs future analysis. Given the quantity of proteins and metabolites probably forming *in vivo* complexes with the different NDPKs, the latter undoubtedly represent hubs in nucleotide homeostasis, making them particularly interesting for any interactomics study.

## Supplementary data

Supplementary data are available at *JXB* online.

Table S1. Vectors used in this study.

Table S2. Oligonucleotides used in this study.

Table S3. Transgenic lines used in this study.

Table S4. Information from the MaxQuant output table ‘proteinGroups.txt’.

Table S5. MaxQuant output table ‘parameters.txt’.

Table S6. MaxQuant output table ‘summary.txt’.

Table S7. Proteins found in the empty vector AP samples.

Table S8. Metabolites found in the empty vector (EV) and blank AP samples.

Table S9. Proteins found in the AP samples, in which NDPK1, NDPK2, or NDPK3 were used as a bait.

Table S10. Metabolites found in the AP samples, in which NDPK1, NDPK2 or NDPK3 were used as a bait.

Fig. S1. Western blot analysis of NDPK–TAP protein expression in transgenic *A. thaliana* cell cultures and eluate fraction of AP following TEV cleavage.

Fig. S2. Reproducibility of replicates presented as a boxplot (R Core Team, 2014).

Fig. S3A. Selected MS/MS spectra and score values indicating quality of identification of the glutathionylation site of NDPK1.

## Supplementary Material

Supplementary Figures S1-S3

Supplementary Tables S1-S10
