# Supplementary material for: Affinity purification with metabolomic and proteomic analysis unravels diverse roles of nucleoside diphosphate kinases
Source: J Exp Bot. 2017 Jun 6;68(13):3487–99. doi: 10.1093/jxb/erx183 (PMC5853561; doi:10.1093/jxb/erx183)
Supplement: Supplementary Figures S1-S3 [file erx183_suppl_supplementary_figures_s1_s3.pdf]

Affinity purification with metabolomic and proteomic analysis unravels diverse roles of nucleoside diphosphate kinases

Running title: *New insight into interactome of plant NDPKs.*

Marcin Luzarowski[1], Monika Kosmacz[1], Ewelina Sokolowska[1], Weronika Jasińska[1], Lothar Willmitzer[1,2], Daniel Veyel[1,2], Aleksandra Skirycz[1,2*]

Figure S1. Western blot analysis of NDPK-TAP protein expression in transgenic *A. thaliana* cell cultures (top panel) **and eluate fraction of AP following TEV cleavage** (lower panel)**.** Predicted size of proteins with a TAP tag (G-protein/streptavidin-binding domain): NDPK1-TAP: 40 kDa, NDPK2-TAP: 46 kDa, NDPK3-TAP: 47 kDa, TAP tag: 21 kDa (top panel). NDPK-TAP stands for C-terminal fusion, while TAP-NDPK for N-terminal fusion. Predicted size of proteins with the S part of the tag (streptavidin-binding domain): streptavidin-binding domain: 7 kDa, NDPK1-S: 26 kDa, NDPK2-S: 32 kDa, NDPK3-S: 32 kDa (lower panel). Western blot analysis was performed to confirm the presence of the tagged proteins. Note that since the loadings were unequal, the results cannot be used to compare protein quantity.

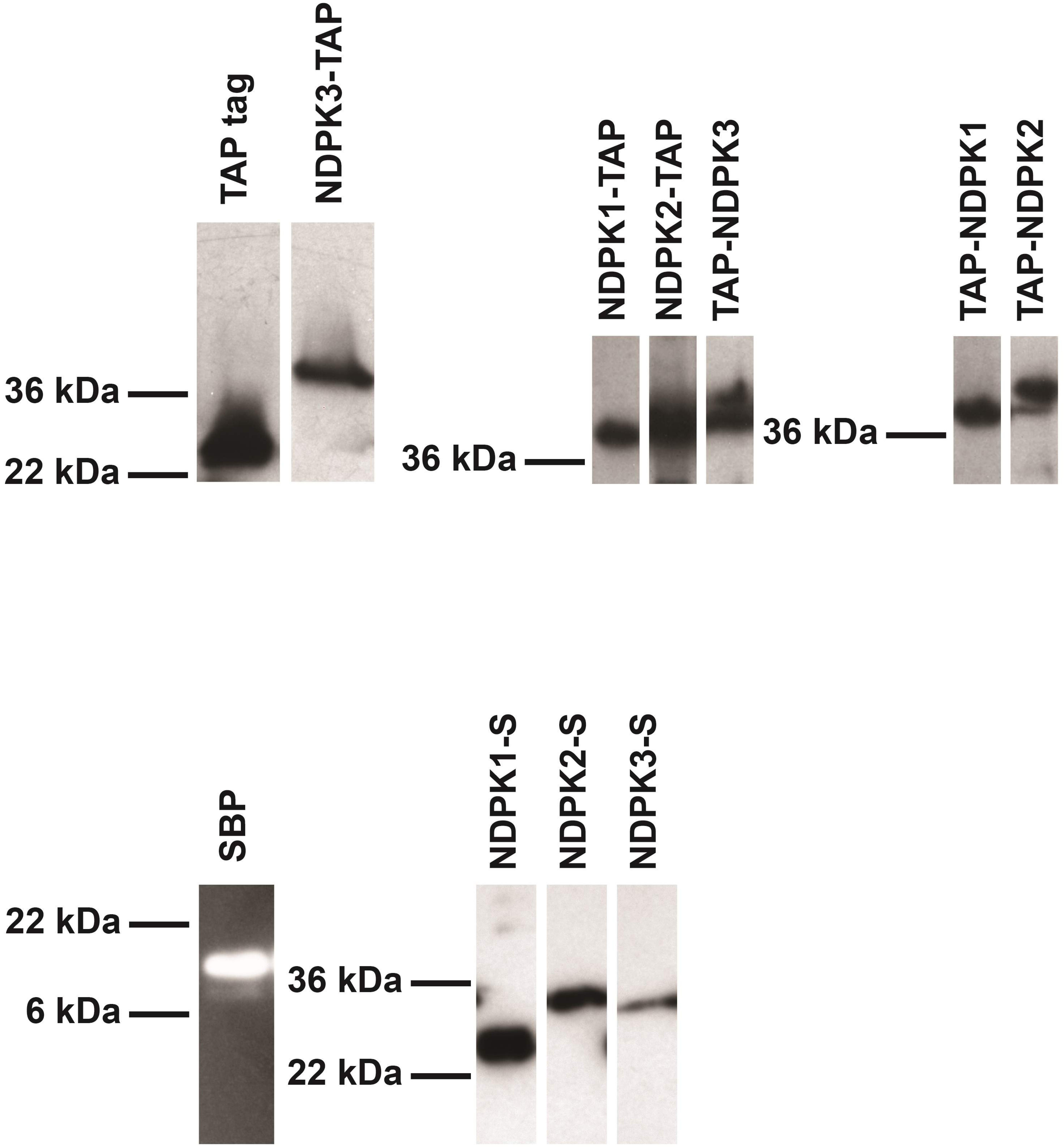
TAP tag
NDPK3-TAP
36 kDa
22 kDa
NDPK1-TAP
NDPK2-TAP
TAP-NDPK3
36 kDa
TAP-NDPK1
TAP-NDPK2
36 kDa
SBP
22 kDa
6 kDa
NDPK1-S
NDPK2-S
NDPK3-S
36 kDa
22 kDa

Figure S2. Boxplot analysis of protein RAW intensity among different samples. EV stand for empty-vector control, NDPKx stand for overexpressed protein, N stands for N-terminal TAP fusion, C stands for C-terminal TAP fusion, 1–3 stand for replicates.

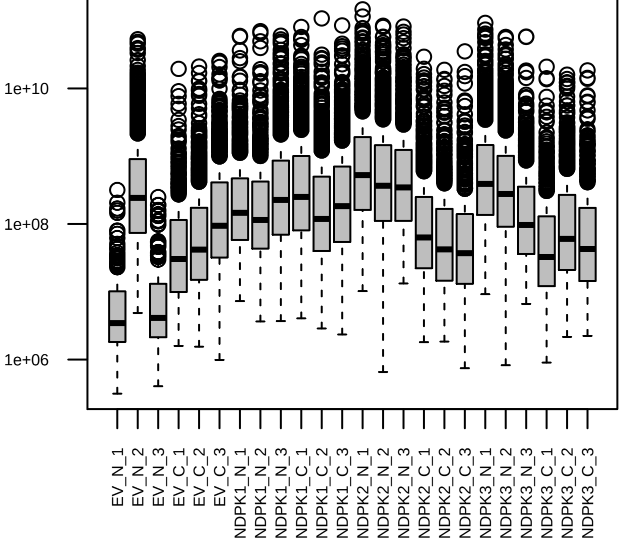

1e+10
1e+08
1e+06
EV_N_1
EV_N_2
EV_N_3
EV_C_1
EV_C_2
EV_C_3
NDPK1_N_1
NDPK1_N_2
NDPK1_N_3
NDPK1_C_1
NDPK1_C_2
NDPK1_C_3
NDPK2_N_1
NDPK2_N_2
NDPK2_N_3
NDPK2_C_1
NDPK2_C_2
NDPK2_C_3
NDPK3_N_1
NDPK3_N_2
NDPK3_N_3
NDPK3_C_1
NDPK3_C_2
NDPK3_C_3

Figure S3. MS/MS spectra indicating quality of identification of glutathionylation site of NDPK1. (A-B) Diamide and GSH treatment, (C-D) GSSG treatment, (E-F) H_2O_2 and GSH treatment, (G-H) GSH treatment.

Search parameters

**Type of search**	: MS/MS Ion Search
**Enzyme**	: Trypsin
**Variable modifications**	: Acetyl (N-term), Carbamidomethyl (C), Glutathione (C), Oxidation (M), Phospho (ST), Phospho (Y)
**Mass values**	: Monoisotopic
**Protein mass**	: Unrestricted
**Peptide mass tolerance**	: ± 5 ppm
**Fragment mass tolerance**	: ± 10 ppm
**Max missed cleavages**	: 2
**Instrument type**	: ESI-FTICR

A) GLIGEVICR - Diamide and GSH treatment

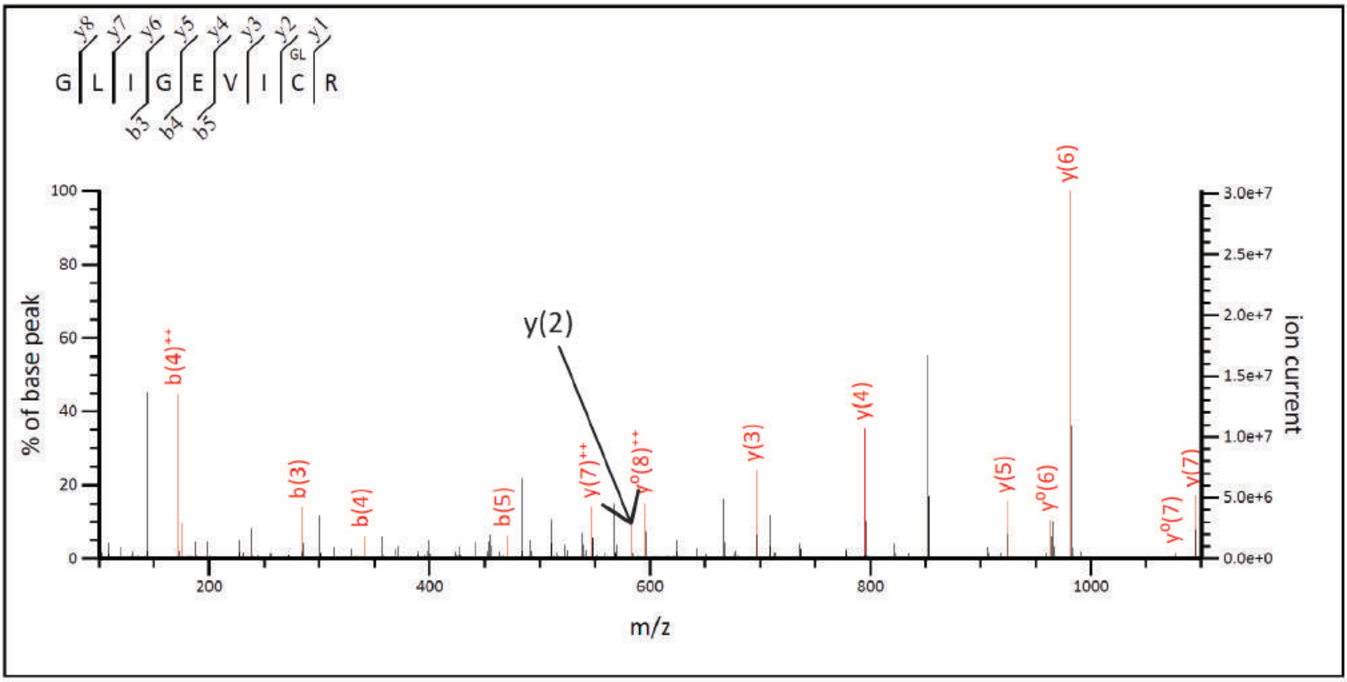


```
Monoisotopic mass of neutral peptide Mr(calc): 1263.5951
Variable modifications:
C8      : Glutathione (C)
Ions Score: 30   Expect: 0.0015
Matches : 16/64 fragment ions using 41 most intense peaks
```

#	b	b^{++}	b^{0}	b^{0++}	Seq.	y	y^{++}	y^{*}	y^{*++}	y^{0}	y^{0++}	#
1	58.0287	29.5180			G							9
2	171.1128	86.0600			L	1207.5810	604.2941	1190.5544	595.7808	1189.5704	595.2888	8
3	284.1969	142.6021			I	1094.4969	547.7521	1077.4703	539.2388	1076.4863	538.7468	7
4	341.2183	171.1128			G	981.4128	491.2101	964.3863	482.6968	963.4023	482.2048	6
5	470.2609	235.6341	452.2504	226.6288	E	924.3914	462.6993	907.3648	454.1860	906.3808	453.6940	5
6	569.3293	285.1683	551.3188	276.1630	V	795.3488	398.1780	778.3222	389.6647			4
7	682.4134	341.7103	664.4028	332.7051	I	696.2804	348.6438	679.2538	340.1305			3
8	1090.4907	545.7490	1072.4802	536.7437	C	583.1963	292.1018	566.1697	283.5885			2
9					R	175.1190	88.0631	158.0924	79.5498			1

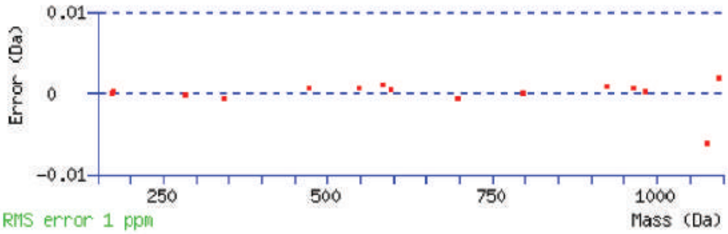

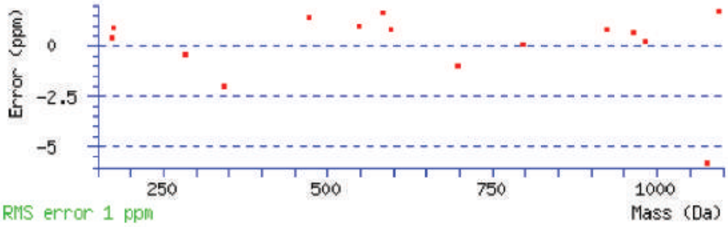

Score	Mr(calc)	Delta	Sequence
30.0	1263.5951	0.0026	GLIGEVICR

B) GLIGEVICRFEK - Diamide and GSH treatment

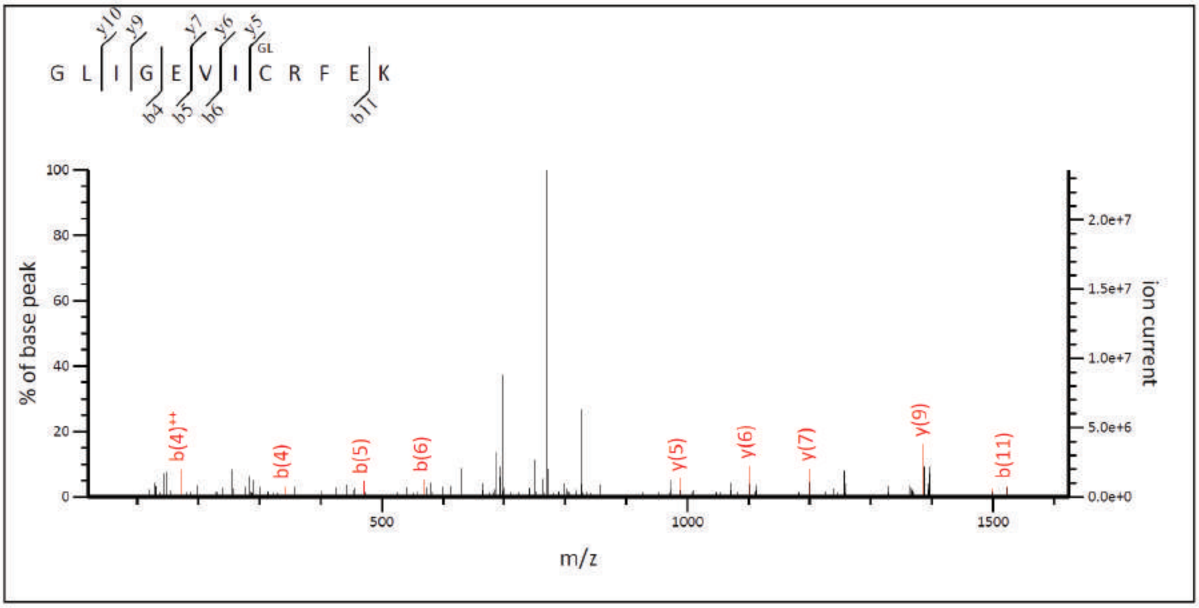


```
Monoisotopic mass of neutral peptide Mr(calc): 1667.8011
Variable modifications:
C8     : Glutathione (C)
Ions Score: 36  Expect: 0.00098
Matches : 11/106 fragment ions using 15 most intense peaks
```

#	b	b++	b*	b*++	b0	b0++	Seq.	y	y++	y*	y*++	y0	y0++	#
1	58.0287	29.5180					G							12
2	*171.1128*	86.0600					L	1611.7869	806.3971	1594.7604	797.8838	1593.7764	797.3918	11
3	284.1969	142.6021					I	*1498.7029*	749.8551	1481.6763	741.3418	1480.6923	740.8498	10
4	*341.2183*	171.1128					G	*1385.6188*	693.3130	1368.5922	684.7998	1367.6082	684.3078	9
5	*470.2609*	235.6341			452.2504	226.6288	E	1328.5973	664.8023	1311.5708	656.2890	1310.5868	655.7970	8
6	*569.3293*	285.1683			551.3188	276.1630	V	*1199.5547*	600.2810	1182.5282	591.7677	1181.5442	591.2757	7
7	682.4134	341.7103			664.4028	332.7051	I	*1100.4863*	550.7468	1083.4598	542.2335	1082.4758	541.7415	6
8	1090.4907	545.7490			1072.4802	536.7437	C	*987.4023*	494.2048	970.3757	485.6915	969.3917	485.1995	5
9	1246.5919	623.7996	1229.5653	615.2863	1228.5813	614.7943	R	579.3249	290.1661	562.2984	281.6528	561.3144	281.1608	4
10	1393.6603	697.3338	1376.6337	688.8205	1375.6497	688.3285	F	423.2238	212.1155	406.1973	203.6023	405.2132	203.1103	3
11	*1522.7029*	761.8551	1505.6763	753.3418	1504.6923	752.8498	E	276.1554	138.5813	259.1288	130.0681	258.1448	129.5761	2
12							K	147.1128	74.0600	130.0863	65.5468			1

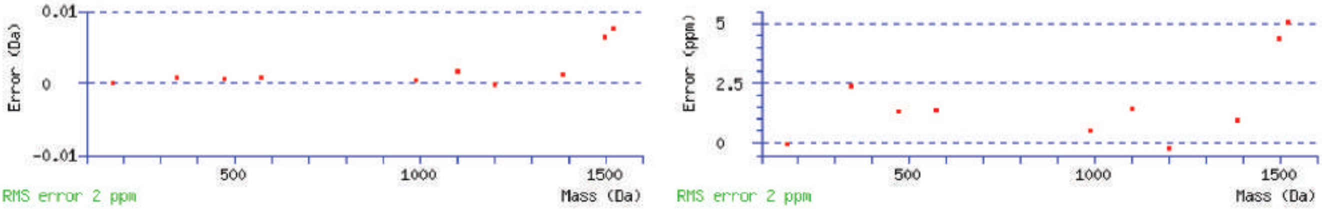

Score	Mr(calc)	Delta	Sequence
36.0	1667.8011	0.0034	GLIGEVICRFEK
3.6	1667.8106	-0.0061	GILWCGDVTVERLK
3.4	1667.8106	-0.0062	LGLGHVVYVSGLSCK
3.4	1667.8106	-0.0062	LGLGHVVYVSGLSCK
3.4	1667.8106	-0.0062	LGLGHVVYVSGLSCK

C) GLIGEVICR - GSSG treatment

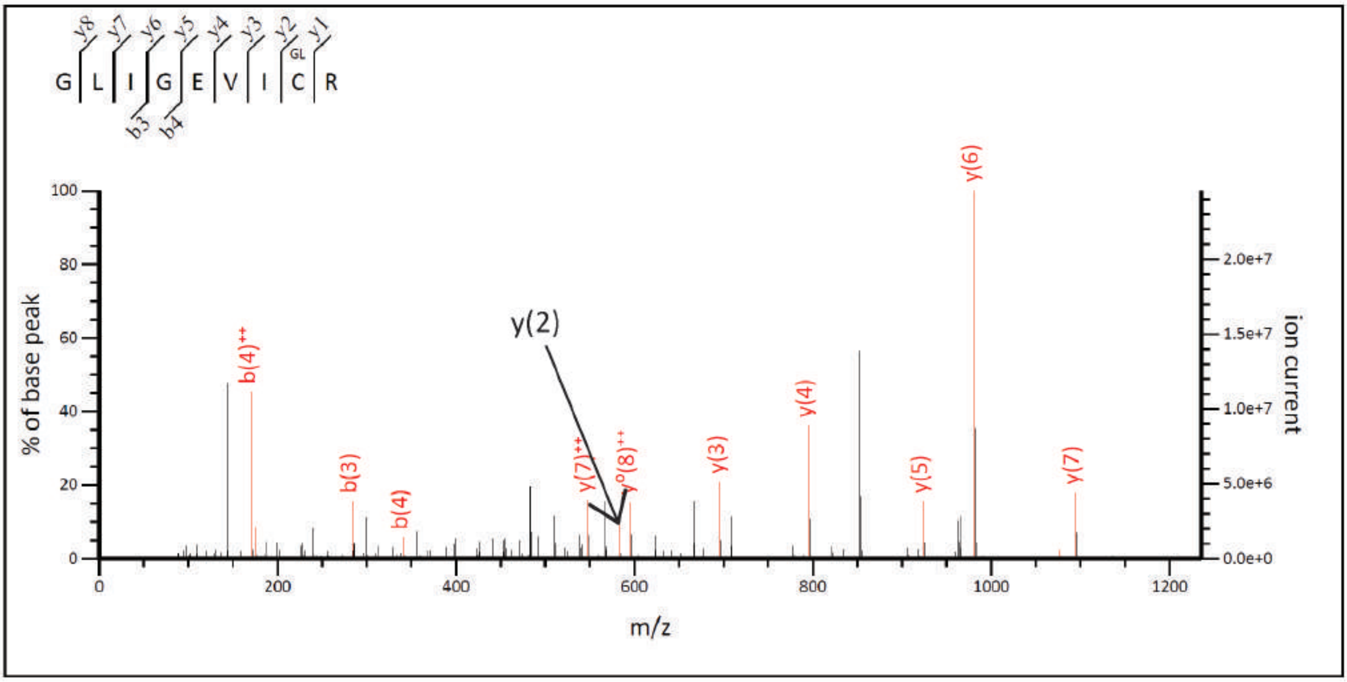


```
Monoisotopic mass of neutral peptide Mr(calc): 1263.5951
Variable modifications:
C8     : Glutathione (C)
Ions Score: 28  Expect: 0.0025
Matches : 15/64 fragment ions using 42 most intense peaks
```

#	b	b^{++}	b^0	b^{0++}	Seq.	y	y^{++}	y*	y^{*++}	y^0	y^{0++}	#
1	58.0287	29.5180			G							9
2	171.1128	86.0600			L	1207.5810	604.2941	1190.5544	595.7808	1189.5704	595.2888	8
3	284.1969	142.6021			I	1094.4969	547.7521	1077.4703	539.2388	1076.4863	538.7468	7
4	341.2183	171.1128			G	981.4128	491.2101	964.3863	482.6968	963.4023	482.2048	6
5	470.2609	235.6341	452.2504	226.6288	E	924.3914	462.6993	907.3648	454.1860	906.3808	453.6940	5
6	569.3293	285.1683	551.3188	276.1630	V	795.3488	398.1780	778.3222	389.6647			4
7	682.4134	341.7103	664.4028	332.7051	I	696.2804	348.6438	679.2538	340.1305			3
8	1090.4907	545.7490	1072.4802	536.7437	C	583.1963	292.1018	566.1697	283.5885			2
9					R	175.1190	88.0631	158.0924	79.5498			1

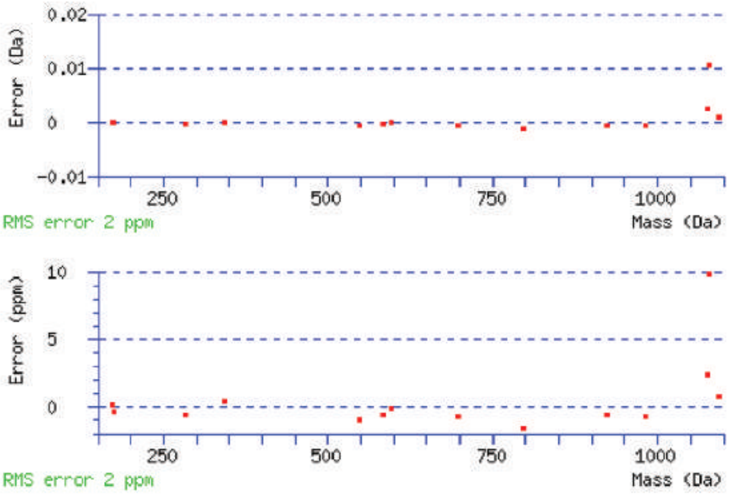

Score	Mr(calc)	Delta	Sequence
27.8	1263.5951	0.0026	GLIGEVICR

D) GLIGEVICRFEK - GSSG treatment

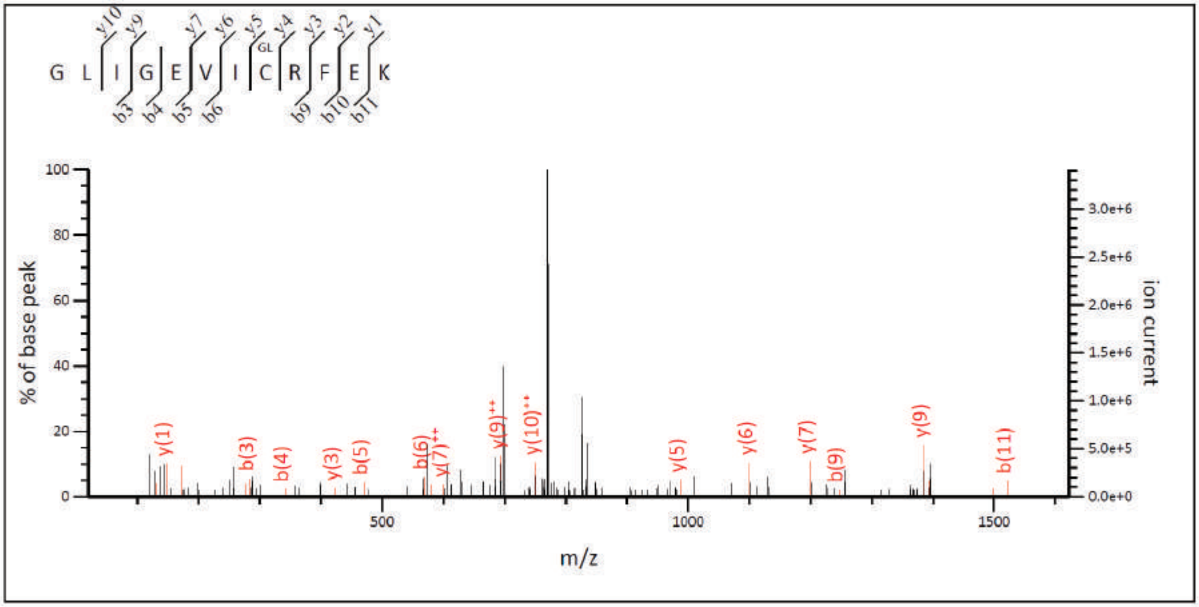

Monoisotopic mass of neutral peptide Mr(calc): 1667.8011
Variable modifications:
C8 : Glutathione (C)
Ions Score: 17 **Expect:** 0.025
Matches : 22/106 fragment ions using 91 most intense peaks

#	b	b^{++}	b*	b^{*++}	b^0	b^{0++}	Seq.	y	y^{++}	y*	y^{*++}	y^0	y^{0++}	#
1	58.0287	29.5180					G							12
2	**171.1128**	86.0600					L	1611.7869	806.3971	1594.7604	797.8838	1593.7764	797.3918	11
3	**284.1969**	142.6021					I	***1498.7029***	**749.8551**	1481.6763	741.3418	1480.6923	740.8498	10
4	**341.2183**	**171.1128**					G	***1385.6188***	**693.3130**	1368.5922	684.7998	1367.6082	684.3078	9
5	**470.2609**	235.6341			452.2504	226.6288	E	1328.5973	664.8023	1311.5708	656.2890	1310.5868	655.7970	8
6	**569.3293**	285.1683			551.3188	276.1630	V	***1199.5547***	**600.2810**	1182.5282	591.7677	1181.5442	591.2757	7
7	682.4134	341.7103			664.4028	332.7051	I	***1100.4863***	550.7468	1083.4598	542.2335	1082.4758	541.7415	6
8	1090.4907	545.7490			1072.4802	536.7437	C	***987.4023***	494.2048	970.3757	485.6915	969.3917	485.1995	5
9	**1246.5919**	623.7996	1229.5653	615.2863	1228.5813	614.7943	R	***579.3249***	290.1661	562.2984	281.6528	561.3144	281.1608	4
10	**1393.6603**	697.3338	1376.6337	688.8205	1375.6497	688.3285	F	***423.2238***	212.1155	406.1973	203.6023	405.2132	203.1103	3
11	**1522.7029**	761.8551	1505.6763	753.3418	1504.6923	752.8498	E	***276.1554***	138.5813	259.1288	130.0681	258.1448	129.5761	2
12							K	***147.1128***	74.0600	130.0863	65.5468			1

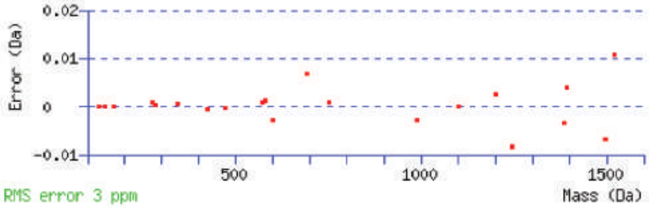

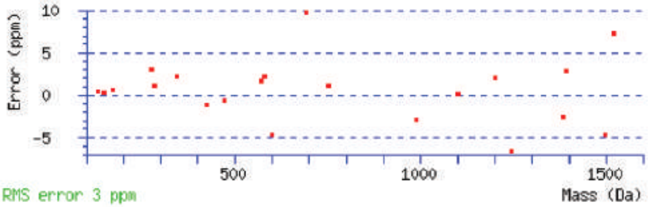

Score	Mr(calc)	Delta	Sequence
17.0	1667.8011	0.0069	GLIGEVICRFEK

E) GLIGEVICR - H_2O_2 and GSH treatment

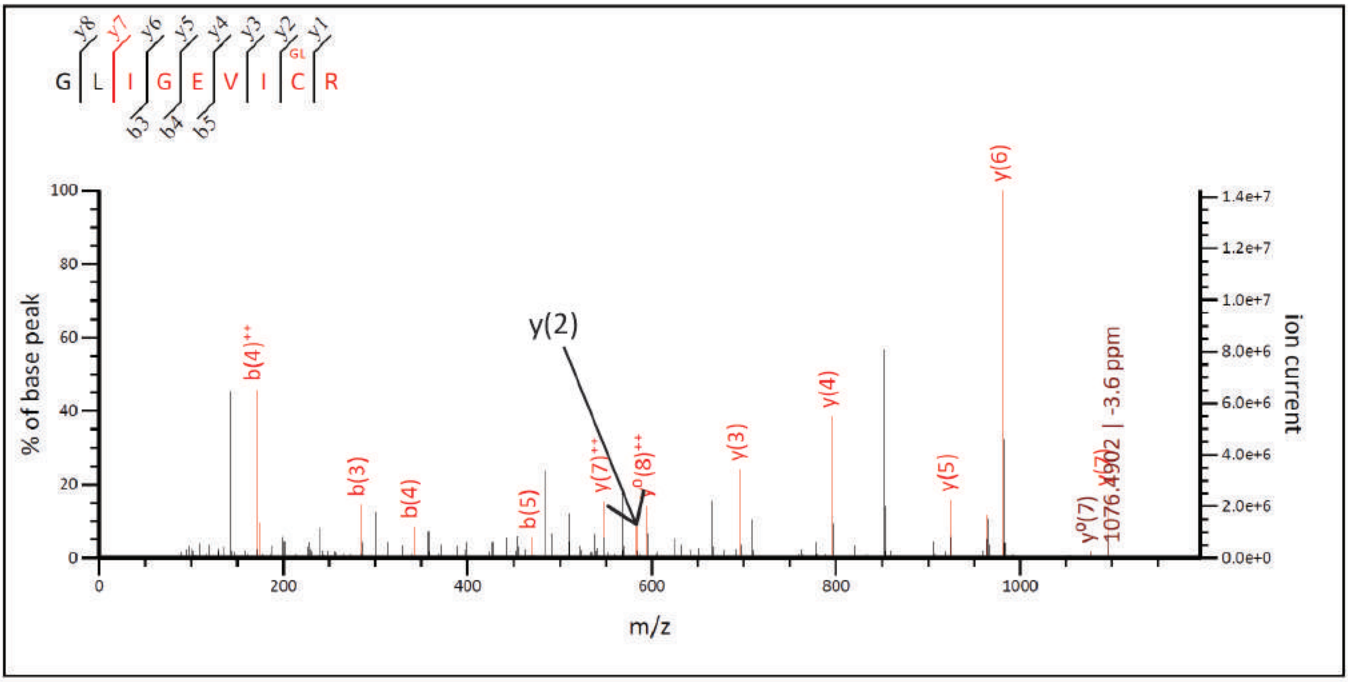

Monoisotopic mass of neutral peptide Mr(calc): 1263.5951
Variable modifications:
C8 : Glutathione (C)
Ions Score: 29 **Expect:** 0.0028
Matches : 16/64 fragment ions using 42 most intense peaks

#	b	b++	b0	b0++	Seq.	y	y++	y*	y*++	y0	y0++	#
1	58.0287	29.5180			G							9
2	**171.1128**	86.0600			L	1207.5810	604.2941	1190.5544	595.7808	1189.5704	595.2888	8
3	**284.1969**	142.6021			I	***1094.4969***	**547.7521**	1077.4703	539.2388	1076.4863	538.7468	7
4	**341.2183**	**171.1128**			G	***981.4128***	491.2101	964.3863	482.6968	963.4023	482.2048	6
5	**470.2609**	235.6341	452.2504	226.6288	E	***924.3914***	462.6993	907.3648	454.1860	906.3808	453.6940	5
6	569.3293	285.1683	551.3188	276.1630	V	***795.3488***	398.1780	778.3222	389.6647			4
7	682.4134	341.7103	664.4028	332.7051	I	***696.2804***	348.6438	679.2538	340.1305			3
8	1090.4907	545.7490	1072.4802	536.7437	C	***583.1963***	292.1018	566.1697	283.5885			2
9					R	***175.1190***	88.0631	158.0924	79.5498			1

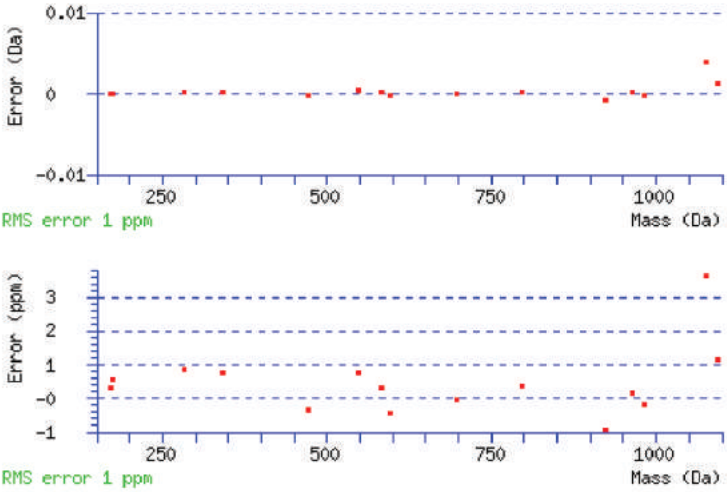

Score	Mr(calc)	Delta	Sequence
29.3	1263.5951	0.0026	GLIGEVICR
2.0	1263.5974	0.0003	KLFIEFMSK

F) GLIGEVICRFEK - H_2O_2 and GSH treatment

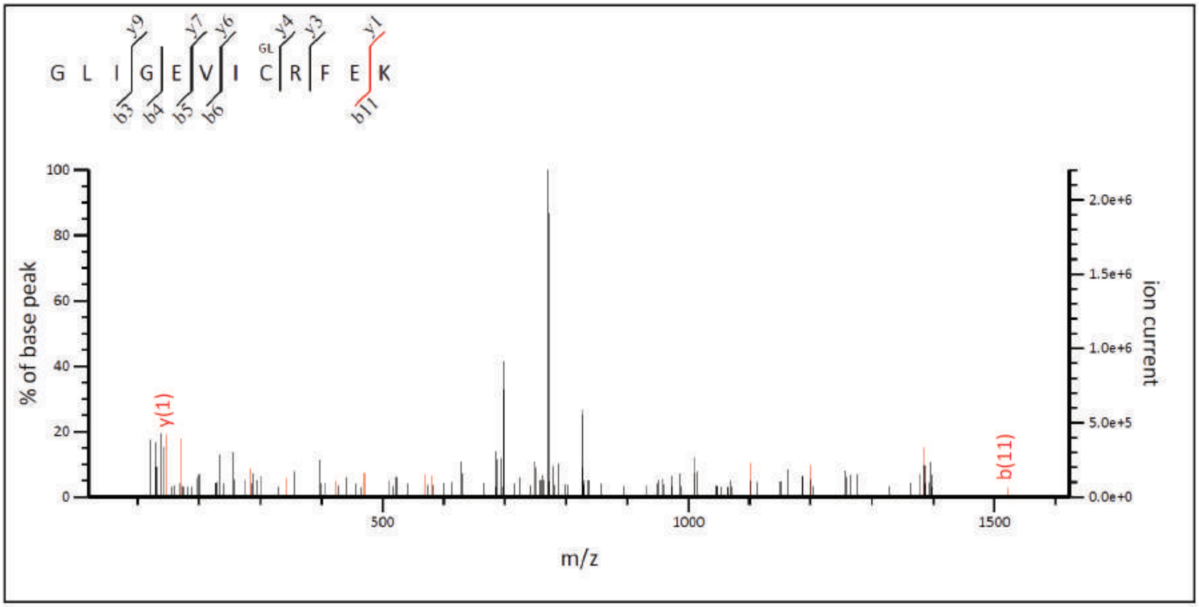

Monoisotopic mass of neutral peptide Mr(calc): 1667.8011
Variable modifications:
C8 : Glutathione (C)
Ions Score: 17 **Expect:** 0.04
Matches : 13/106 fragment ions using 40 most intense peaks

#	b	b^{++}	b^{*}	b^{*++}	b^{0}	b^{0++}	Seq.	y	y^{++}	y^{*}	y^{*++}	y^{0}	y^{0++}	#
1	58.0287	29.5180					G							12
2	171.1128	86.0600					L	1611.7869	806.3971	1594.7604	797.8838	1593.7764	797.3918	11
3	284.1969	142.6021					I	1498.7029	749.8551	1481.6763	741.3418	1480.6923	740.8498	10
4	341.2183	171.1128					G	1385.6188	693.3130	1368.5922	684.7998	1367.6082	684.3078	9
5	470.2609	235.6341			452.2504	226.6288	E	1328.5973	664.8023	1311.5708	656.2890	1310.5868	655.7970	8
6	569.3293	285.1683			551.3188	276.1630	V	1199.5547	600.2810	1182.5282	591.7677	1181.5442	591.2757	7
7	682.4134	341.7103			664.4028	332.7051	I	1100.4863	550.7468	1083.4598	542.2335	1082.4758	541.7415	6
8	1090.4907	545.7490			1072.4802	536.7437	C	987.4023	494.2048	970.3757	485.6915	969.3917	485.1995	5
9	1246.5919	623.7996	1229.5653	615.2863	1228.5813	614.7943	R	579.3249	290.1661	562.2984	281.6528	561.3144	281.1608	4
10	1393.6603	697.3338	1376.6337	688.8205	1375.6497	688.3285	F	423.2238	212.1155	406.1973	203.6023	405.2132	203.1103	3
11	1522.7029	761.8551	1505.6763	753.3418	1504.6923	752.8498	E	276.1554	138.5813	259.1288	130.0681	258.1448	129.5761	2
12							K	147.1128	74.0600	130.0863	65.5468			1

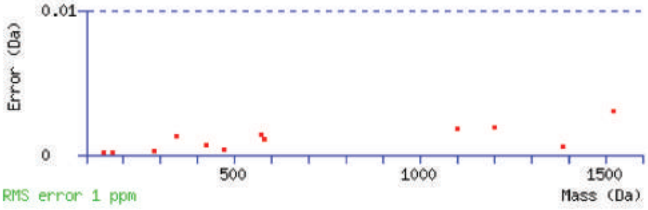

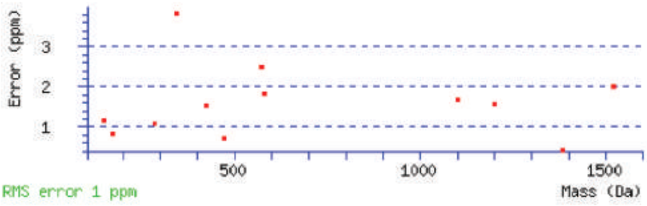

Score	Mr(calc)	Delta	Sequence
16.7	1667.8011	0.0034	GLIGEVICRFEK
1.6	1667.8066	-0.0021	LGEPLRRMSLESGK

G) GLIGEVICR - GSH treatment

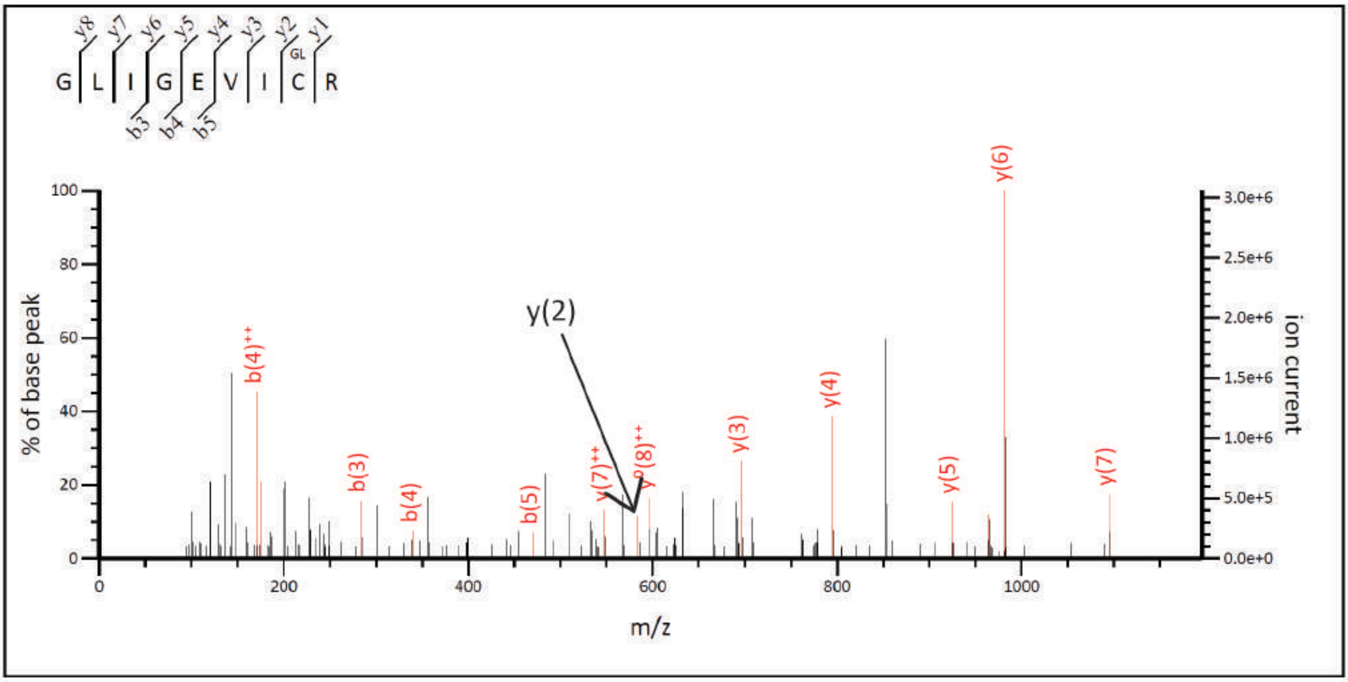

Monoisotopic mass of neutral peptide Mr(calc): 1263.5951
Variable modifications:
C8 : Glutathione (C)
Ions Score: 26 **Expect:** 0.0035
Matches : 15/64 fragment ions using 41 most intense peaks

#	b	b^{++}	b^{0}	b^{0++}	Seq.	y	y^{++}	y*	y^{*++}	y^{0}	y^{0++}	#
1	58.0287	29.5180			G							9
2	**171.1128**	86.0600			L	1207.5810	604.2941	1190.5544	595.7808	1189.5704	595.2888	8
3	**284.1969**	142.6021			I	***1094.4969***	**547.7521**	1077.4703	539.2388	1076.4863	538.7468	7
4	**341.2183**	**171.1128**			G	***981.4128***	491.2101	964.3863	482.6968	963.4023	482.2048	6
5	**470.2609**	235.6341	452.2504	226.6288	E	***924.3914***	462.6993	907.3648	454.1860	906.3808	453.6940	5
6	569.3293	285.1683	551.3188	276.1630	V	***795.3488***	398.1780	778.3222	389.6647			4
7	682.4134	341.7103	664.4028	332.7051	I	***696.2804***	348.6438	679.2538	340.1305			3
8	1090.4907	545.7490	1072.4802	536.7437	C	***583.1963***	292.1018	566.1697	283.5885			2
9					R	***175.1190***	88.0631	158.0924	79.5498			1

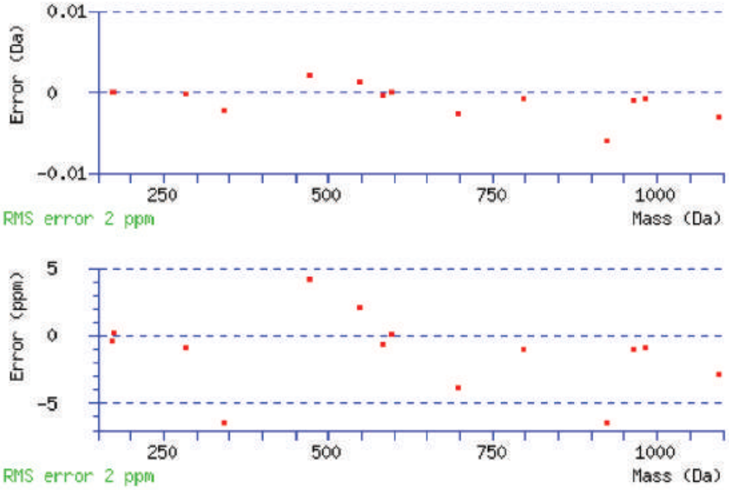

Score	Mr(calc)	Delta	Sequence
26.2	1263.5951	0.0026	GLIGEVICR

H) GLIGEVICRFEK - GSH treatment

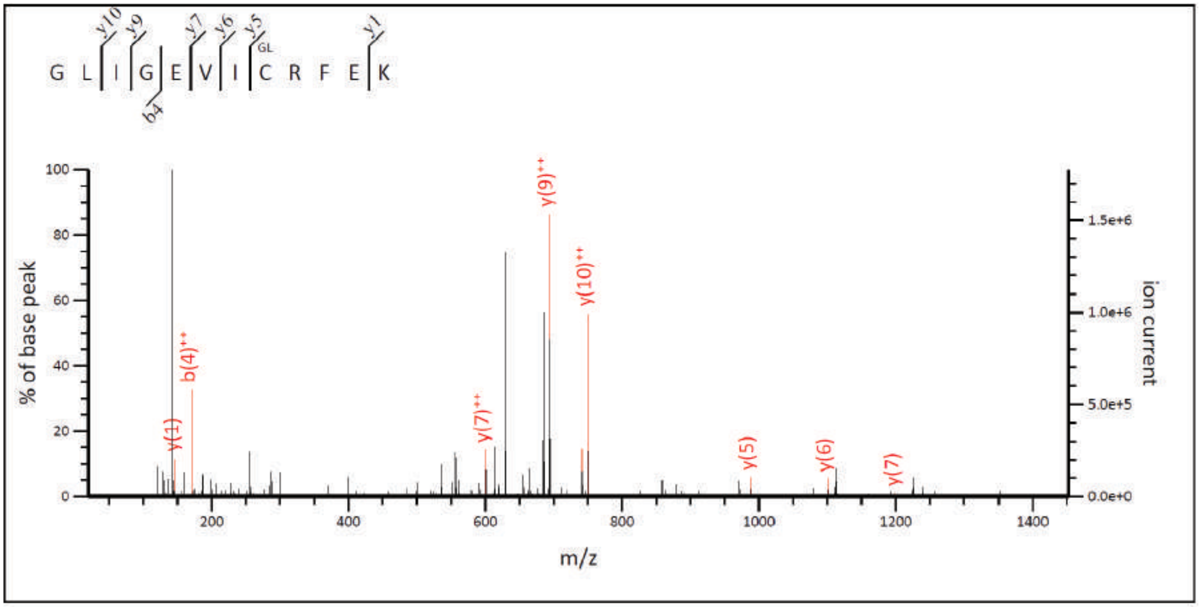

Monoisotopic mass of neutral peptide Mr(calc): 1667.8011
Variable modifications:
C8 : Glutathione (C)
Ions Score: 5 **Expect:** 0.35
Matches : 10/106 fragment ions using 37 most intense peaks

#	b	b++	b*	b*++	b0	b0++	Seq.	y	y++	y*	y*++	y0	y0++	#
1	58.0287	29.5180					G							12
2	**171.1128**	86.0600					L	1611.7869	806.3971	1594.7604	797.8838	1593.7764	797.3918	11
3	284.1969	142.6021					I	1498.7029	***749.8551***	1481.6763	741.3418	1480.6923	740.8498	10
4	341.2183	**171.1128**					G	1385.6188	***693.3130***	1368.5922	684.7998	1367.6082	684.3078	9
5	470.2609	235.6341			452.2504	226.6288	E	1328.5973	664.8023	1311.5708	656.2890	1310.5868	655.7970	8
6	569.3293	285.1683			551.3188	276.1630	V	***1199.5547***	***600.2810***	1182.5282	591.7677	1181.5442	591.2757	7
7	682.4134	341.7103			664.4028	332.7051	I	***1100.4863***	550.7468	1083.4598	542.2335	1082.4758	541.7415	6
8	1090.4907	545.7490			1072.4802	536.7437	C	***987.4023***	494.2048	970.3757	485.6915	969.3917	485.1995	5
9	1246.5919	623.7996	1229.5653	615.2863	1228.5813	614.7943	R	579.3249	290.1661	562.2984	281.6528	561.3144	281.1608	4
10	1393.6603	697.3338	1376.6337	688.8205	1375.6497	688.3285	F	423.2238	212.1155	406.1973	203.6023	405.2132	203.1103	3
11	1522.7029	761.8551	1505.6763	753.3418	1504.6923	752.8498	E	276.1554	138.5813	259.1288	130.0681	258.1448	129.5761	2
12							K	***147.1128***	74.0600	130.0863	65.5468			1

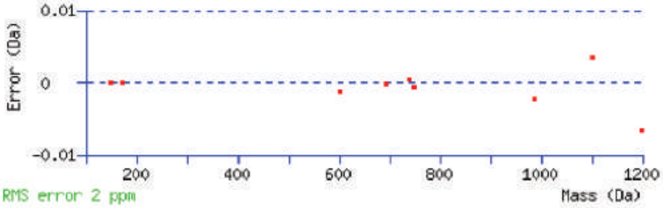

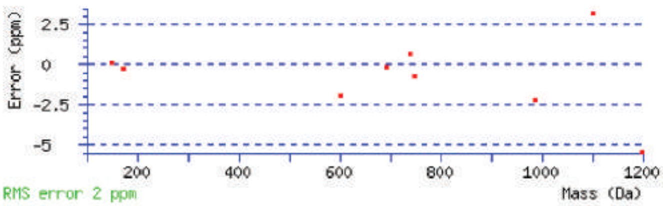

Score	Mr(calc)	Delta	Sequence
4.9	1667.8011	0.0015	GLIGEVICRFEK
